# Expression of *SAA1*, *SAA2* and *SAA4* genes in human primary monocytes and monocyte-derived macrophages

**DOI:** 10.1371/journal.pone.0217005

**Published:** 2019-05-17

**Authors:** Claire Jumeau, Fawaz Awad, Eman Assrawi, Laetitia Cobret, Philippe Duquesnoy, Irina Giurgea, Dominique Valeyre, Gilles Grateau, Serge Amselem, Jean-François Bernaudin, Sonia-Athina Karabina

**Affiliations:** 1 Sorbonne Université, INSERM, UMR_S 933, Assistance Publique Hôpitaux de Paris, Hôpital Trousseau, Service de Génétique et d’Embryologie médicale, Paris, France; 2 Assistance Publique Hôpitaux de Paris, Hôpital Avicenne, Service de Pneumologie, Bobigny, France; 3 Université Paris 13, INSERM UMR 1272, Laboratoire ‘Hypoxie & Poumon’, Bobigny, France; 4 Assistance Publique Hôpitaux de Paris, Hôpital Tenon, Service de médecine interne, Paris, France; University of California San Diego, UNITED STATES

## Abstract

Circulating serum amyloid A (SAA) is increased in various inflammatory conditions. The human SAA protein family comprises the acute phase SAA1/SAA2, known to activate a large set of innate and adaptive immune cells, and the constitutive SAA4. The liver synthesis of SAA1/SAA2 is well-established but there is still an open debate on extrahepatic SAA expression especially in macrophages. We aimed to investigate the ability of human primary monocytes and monocyte-derived macrophages to express *SAA1*, *SAA2* and *SAA4* at both the transcriptional and protein levels, as previous studies almost exclusively dealt with monocytic cell lines. Monocytes and derived macrophages from healthy donors were stimulated under various conditions. In parallel with *SAA*, pro-inflammatory *IL1A*, *IL1B* and *IL6* cytokine expression was assessed. While LPS alone was non-effective, a combined LPS/dexamethasone treatment induced *SAA1* and to a lesser extent *SAA2* transcription in human monocytes and macrophages. In contrast, as expected, pro-inflammatory cytokine expression was strongly induced following stimulation with LPS, an effect which was dampened in the presence of dexamethasone. Furthermore, in monocytes polarized towards a pro-inflammatory M1 phenotype, *SAA* expression in response to LPS/dexamethasone was potentiated; a result mainly seen for *SAA1*. However, a major discrepancy was observed between *SAA* mRNA and intracellular protein levels under the experimental conditions used. Our results demonstrate that human monocytes and macrophages can express *SAA* genes, mainly *SAA1* in response to an inflammatory environment. While SAA is considered as a member of a large cytokine network, its expression in the monocytes-macrophages in response to LPS-dexamethasone is strikingly different from that observed for classic pro-inflammatory cytokines. As monocytes-macrophages are major players in chronic inflammatory diseases, it may be hypothesized that SAA production from macrophages may contribute to the local inflammatory microenvironment, especially when macrophages are compactly organized in granulomas as in sarcoidosis.

## Introduction

In humans, serum amyloid A (SAA) is encoded by four *SAA* genes, mapping to a 150-kb region of chromosome 11p15.1 [1–3]. *SAA1* and *SAA2* are co-ordinately regulated acute phase response genes, sharing > 95% nucleotide identity [4]. *SAA3*, initially considered as a non-transcribed gene, [5], has since been detected in mammary gland epithelial cell lines following stimulation with prolactin or lipopolysaccharide (LPS) [6]. As for *SAA4*, it is constitutively expressed [2,7,8].

The corresponding SAA proteins are highly conserved among species, indicating important biological functions [9]. However, whether SAA1 and SAA2 have distinct roles in physiology and in the pathophysiology of human disorders is still unknown [10]. In response to tissue injury or infection, a dramatic increase in SAA plasma levels (SAA1 and SAA2) is observed; consequently, SAA is considered as a marker of inflammation [11]. During acute phase, high levels of circulating SAA1 and SAA2 displace apolipoprotein A1 from high density lipoprotein (HDL), becoming an HDL-associated apolipoprotein [12–15]. This leads to a loss of the anti-inflammatory properties of HDL [16]. SAA proteins are also the precursors of Amyloid A (AA) fibril formation, responsible for tissue and organ amyloidosis [17,18]. Recombinant human SAA (rhSAA) has been shown to induce chemotactic activity in neutrophils, dendritic cells, monocytes and T lymphocytes [8,11,19–21] and secretion of cytokines [22,23]. These pleiotropic functions of SAA are thought to be mediated through signalling pathways following engagement of cell-surface receptors like the Toll-like and scavenger receptors [24–26]. Overall, based on these effects, SAA are therefore considered as pro-inflammatory proteins. However, SAA is not only an acute inflammation response mediator, but also plays a significant role in the pathogenesis of various chronic diseases at the crossroad of autoimmunity and autoinflammation such as Behcet’s disease [27,28] or sarcoidosis [29,30].

The secretion of SAA by the liver as for all the acute phase proteins is well established. Indeed, hepatic cells, such as the HepG2 cell line secrete SAA following stimulation with pro-inflammatory cytokines like interleukin (IL) IL1β, IL6 and tumor necrosis factor alpha (TNFα) [31,32]. However, the possibility that extrahepatic sites of SAA synthesis exist is supported by expression of *SAA* in epithelial components of a variety of normal tissues [33]. Particularly challenging is the potential extrahepatic synthesis of SAA by immune cells. Supportive to this hypothesis is the detection of *SAA* mRNA by *in situ* hybridization (ISH) in macrophage-derived foam cells present in atherosclerotic lesions [34–36]. Furthermore, SAA proteins have been detected by immunohistochemistry (IHC) in macrophage accumulation areas in rheumatoid arthritis joints [37,38] and in macrophage-derived cells constituting the main components of epithelioid and giant cell granulomas in sarcoidosis [29]. It has been suggested that a potential local production of SAA by macrophage-derived epithelioid cells can lead to granuloma formation and maintenance [39–41]. However, although the localization of SAA in macrophages from various inflammatory conditions [29,33,42–44] suggested a possible local production of SAA by macrophages at sites of inflammation, it is still unclear whether the intracellular SAA observed by IHC in macrophages is a result of internalization of the circulating liver-produced SAA or is due to expression by tissue macrophages.

Studies on SAA synthesis by macrophages have almost exclusively dealt with human or rodent cell lines. *SAA* gene transcription has been demonstrated in the human macrophage-like THP1 and U-937 cells treated with LPS-dexamethasone and/or D3 vitamin or PMA (Phorbol Myristate Acetate) [43,45] and in the rodent J-774 cell line following activation by LPS [46]. To our knowledge, there are no extensive studies carried out in naïve human monocytes and macrophages. Therefore, after stimulation by various pro-inflammatory conditions, changes in *SAA1*, *SAA2* and *SAA4* gene expression at both the transcriptional and protein levels were evaluated during maturation of freshly collected human monocytes into macrophages.

## Materials & methods

### Isolation of human peripheral blood monocytes and monocyte-derived macrophages

Peripheral blood mononuclear cells (PBMCs) were isolated from buffy coats, from apparently healthy blood donors, provided by the “*Etablissement Français du Sang*, *((EFS);* convention N°15EFS012 between “INSERM” and EFS; informed consent was obtained from all subjects). PBMCs were isolated using Ficoll density gradient as described previously [47]. Monocytes were selected from PBMCs by 1 h adherence at 37°C and cultured for 24 h in the presence of RPMI 1640 (Cat No. 21875–091, Fischer, Paisley, Scotland, UK) complemented with 10% pooled human serum (HS) (Cat No. C15-021, PAA), L-glutamine (Cat No. 25030–024, Gibco), penicillin and streptomycin (Cat No. 11548876, Gibco) (complete medium). Monocyte-derived macrophages were obtained after 7, 14 and 21 days in culture of adherent monocytes in the presence of complete medium (named respectively 7-day, 14-day and 21-day culture macrophages from here after). Cell viability was tested using the Cell Counting 8 kit (Cat No. 277CK04-05, Tebu Bio).

Alternative to isolation by adherence, monocytes from 2 donors were isolated from PBMCs using CD14 positive selection (130-050-201, Miltenyi Biotec, Bergisch Gladbach, Germany) following the manufacturer’s instructions. Cells were treated with LPS and/or dexamethasone for 24 h in the absence of HS as described below.

### Monocyte polarization

In selected experiments, monocytes were polarized in order to obtain M1 or M2 phenotypes as described before [48]. Briefly, adherent monocytes were treated with either 100 ng/ml interferon (IFN)-γ (M1 phenotype) or 10 ng/ml of IL4 and IL13 (M2 phenotype) for 24 h. Cells were additionally treated with LPS and dexamethasone as described below.

### Monocyte and macrophage treatments

Monocytes immediately after adherence, or 7-day, 14-day, or 21-day culture macrophages were treated with 0.1 or 2 μg/mL LPS or 0.01, 1, 10 μM dexamethasone (Mylan, Saint Priest, France) for 24 h in the absence of HS unless otherwise stated. Results of time- and concentration-dependent experiments are presented in the supplementary data. Based on these results (S1 and S2 Figs), most of the treatments were done, unless otherwise specified, using 100 ng/mL LPS and 1μM dexamethasone. Methylprednisolone (Mylan 20 mg, Saint Priest France), another synthetic glucocorticosteroid was used on selected experiments performed on 7-day culture macrophages from 4 donors.

Monocytes were alternatively treated after washing with 1X PBS with 100 ng/mL IL1β (Cat No. 200-01B, Peprotech), 100 ng/mL IL6 (Cat No. 200–06, Peprotech) and 1μM dexamethasone (Mylan) for 24 h to compare with the results observed with the HepG2 cell line.

Following treatment, cell culture supernatants were collected and stored at -20°C for cytokine measurement by ELISA and cell lysates were used for RNA isolation, immunoprecipitation or western blot analysis.

### Culture and treatment of HepG2 cells

HepG2 cells (a kind gift of Dr Anastasia Tchoukaev, Saint Antoine Hospital, Paris, France) were cultured in presence of DMEM (Cat No. 31966–02, Gibco) complemented with 10% pooled fetal calf serum (FCS) (Cat No. 10500–064, Gibco), L-glutamine (Cat No. 25030–024, Gibco), penicillin and streptomycin (Cat No. 11548876, Gibco) (complete medium). Cells were treated after wash with 1X PBS with 100 ng/mL IL1β (Cat No. 200-01B, Peprotech), 100 ng/mL IL6 (Cat No. 200–06, Peprotech) and 1μM dexamethasone (Mylan) for 24 h in the absence of FCS. Following treatment, cell culture supernatants were collected and stored at -20°C for cytokine measurement by ELISA and cell lysates were used for RNA isolation, immunoprecipitation or western blot analysis.

### Concentration of supernatants

In selected experiments, cell culture supernatants (4 ml) of human monocytes or HepG2 cells were concentrated by centrifugation (2100 g for at least 90 min at room temperature) using Amicon filters with a molecular cut off of 3 kDa (Merck Milipore) until a final volume of 100 μL.

### Reverse transcription and quantitative-PCR (RT-qPCR)

Total RNA from monocytes or macrophages was isolated after treatments using the RNAeasy mini kit (Qiagen) including a DNase step (Qiagen). RNA was then reverse transcribed in the presence of 2.5 mM oligo-dT using the Transcriptor High Fidelity cDNA Synthesis Kit (Roche), following the manufacturer’s instructions. Five ng of cDNA was amplified using the Mesa Blue qPCR MasterMix Plus for SYBR Assay (Eurogentec) in the Light Cycler LC480 (Roche). mRNA expression was normalized to the levels of ribosomal protein L13a, *RPL13A* (NM_012423.2), which was used as housekeeping gene. The glucocorticoid-induced leucine zipper (GILZ) gene expression was used as a marker of glucocorticoid induction [49]. The relative level of expression of a gene between sample 1 (treated) and sample 2 (control) was calculated using the ΔΔCt formula: 2^−(Ct1−Ct *RPL13A* 1)−(Ct2−Ct *RPL13A* 2)^. Normalized Ct values (mean of controls) were used to calculate the gene expression ratio between two samples. Primers (S1 Table) were designed either using the Probe Finder software (http://qpcr.probefinder.com/organism.jsp; Roche Life sciences) or manually for specificity reasons.

### Western blot analysis

Monocytes or macrophages were lysed in 2.5× Laemmli SDS buffer supplemented with 4% beta-mercaptoethanol, 0.02% bromophenol blue and a protease inhibitor cocktail (Roche). Equal amounts of protein were analyzed by 15% SDS-PAGE. Proteins were transferred to PVDF membrane and incubated overnight with a primary mouse anti-human serum amyloid A1 (capture antibody from DuoSet ELISA (DY3019-05 kit R&D, Biotechne, Abingdon, UK) used at 1 μg/ml in 5% BSA-PBS-0.1% Tween) and a secondary anti mouse IgG HRP (1:1000) (Cat No. 7076, Cell Signaling, Danvers, MA, USA). Following wash steps, the blots were revealed using the SuperSignal West Dura Extended Duration substrate (Thermo Scientific) and visualized using a BIORAD camera (Universal Hood II).

### Immunoprecipitation

Immunoprecipitation was done according to the manufacturer’s instructions using our own lysis buffer. Briefly monocytes or 7-day culture macrophages were incubated for 1 hour on ice with 1M Tris pH 7.5, 5M NaCl, 0.5M EDTA buffer, 10% Triton, supplemented with protease (Complete protease inhibitor cocktail, Cat No. 11836145001, Roche, Germany) and phosphatase (from the universal magnetic co-IP kit Cat No. 54002, Active Motif) inhibitors. Lysates were collected and 5 μg of anti-SAA antibody (mouse anti-human serum amyloid A1 capture antibody from DuoSet ELISA DY3019-05 kit R&D, Biotechne) was then added to the sample and incubated overnight at 4°C. 25 μL of magnetic beads were added to each sample the following day, incubated for 1 hour. Five washes were done with the lysis buffer. After the last wash, 20 μL of 2.5x Laemmli-SDS buffer was added to each sample and eluates were used for western blotting.

### ELISA

Cell culture supernatants from human monocytes or macrophages were collected after treatment, centrifuged for 10 minutes at 300 g and kept at -20°C for cytokine measurements. In selected experiments concentrated supernatants were also tested. SAA1 and IL1β were quantified using the DuoSet ELISA kits (Cat No DY3019-05, and DY201-05 respectively, R&D, Biotechne) following manufacturers’ instructions.

### Statistics

Results are presented as the mean ± standard error of the mean (SEM) from experiments performed in cells from at least 4 independent donors unless otherwise specified. The Mann Whitney test was used to evaluate the statistical significance of differences between 2 groups. A value of p<0.05 was considered significant (indicated as * p < 0.05; ** p < 0.01, *** p< 0.0001 or # p < 0.05; ## p < 0.01, ### p< 0.0001, depending on the comparisons made). Graphpad Prism was used to design all figures and statistical tests.

## Results

### Expression of *SAA1*, *SAA2*, *SAA4* and pro-inflammatory cytokines in human adherent monocytes

**Fig 1:**
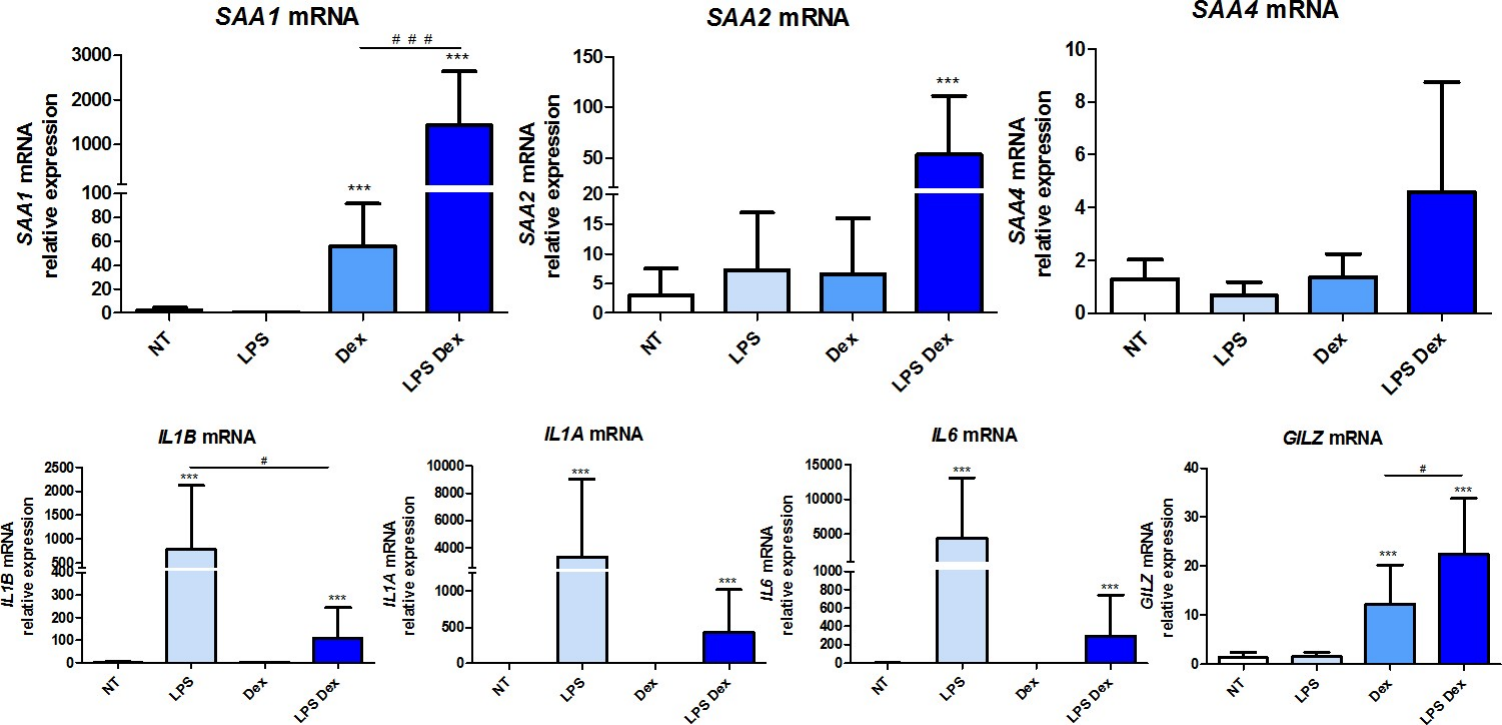
Expression of *SAA1*, *SAA2*, *SAA4* and pro-inflammatory cytokines in human monocytes. mRNA gene expression was measured by RT-qPCR and presented as relative fold change of the non-treated monocytes. Data represent the mean ± standard error of the mean (SEM) of 9 experiments performed in cells isolated from 9 independent donors (buffy coats). Asterisks indicate significant differences as compared to non-treated cells (NT). (Mann Whitney test: * p < 0.05; ** p < 0.01, *** p< 0.0001). # indicates significant differences between the two delimited conditions (Mann Whitney test: # p < 0.05; ## p < 0.01, ### p< 0.0001).

Basal *SAA1*, *SAA2* and *SAA4* mRNA levels were very low (Ct>37) and not significantly modified following stimulation of adherent monocytes with 100 ng/ml LPS (Fig 1). Treatment with 1μM dexamethasone (Dex) alone significantly increased *SAA1* gene expression (Ct from 37 to 32), while no significant change was observed for *SAA2* and *SAA4*. The concentration and incubation periods used for LPS and Dex were defined in separate experiments as described in the methods and presented in the supplement (S1 Appendix, S1 and S2 Figs). Surprisingly, a combined treatment of 100 ng/mL LPS with 1μM Dex induced a major increase in *SAA1* expression (Ct from 37 to 27–28) (p<0.0001) and to a lower extent in *SAA2* expression (Ct from 37 to 33) (p = 0.0003) (Fig 1, upper panel). In contrast, no significant change was observed in *SAA4* expression (Fig 1, upper panel). Under all conditions used *SAA3* expression was not detected.

Expression of the pro-inflammatory cytokines *IL1A*, *IL1B* and *IL6* was used as a positive control of the LPS treatment. As expected, in the presence of LPS alone, mRNA expression of *IL1A*, *IL1B* and *IL6* was significantly induced (p<0.0001), whereas treatment with Dex alone had no effect on their basal expression levels (Fig 1, lower panel). The combined treatment LPS-Dex significantly downregulated *IL1B* gene expression (p = 0.03) as compared to treatment with LPS alone (Fig 1, lower panel).

The basal expression (Ct ~29) of *GILZ* (glucocorticoid induced leucine zipper gene), marker of glucocorticoid action [49], was significantly increased in the presence of Dex (Ct ~25) or LPS-Dex (Ct ~24,5) whereas LPS alone had no impact on its basal expression levels (Ct ~29) (Fig 1, lower panel).

### Expression of *SAA1*, *SAA2*, *SAA4* and pro-inflammatory cytokines in CD14 positively-selected human monocytes

In order to determine whether the technique used for monocyte selection from PBMCs may have an impact on *SAA* gene expression, we isolated monocytes using CD14 positive selection instead of adherence. Basal expression of *SAA* genes in CD14+ monocytes was very low (Ct>37), similar to what was observed in adherent monocytes (S3 Fig). Treatment with Dex alone had no significant impact, while the combination of LPS-Dex induced an increase in *SAA1* expression (Ct from 37 to 28), comparable to that observed in monocytes selected by adherence (S3 Fig). The expression of *SAA2* was also significantly up-regulated (Ct from 38 to 32) in the presence of LPS-Dex although to a lesser extent. *SAA4* expression was variable but under all treatments very low (Ct>35). In the presence of LPS alone, *IL1B* and *IL11A* mRNA expression was induced as expected, whereas treatment with Dex alone had no effect on their basal expression levels. The combined treatment of LPS-Dex down-regulated the LPS-induced gene expression of both *IL1B* and *IL1A* in the 2 donors tested (S3 Fig, lower panel).

### Expression of *SAA1*, *SAA2*, *SAA4* and pro-inflammatory cytokines in human polarized monocytes

We next studied whether monocyte polarization by INF-γ (M1 pro-inflammatory phenotype), or by IL4 and IL13 (M2 phenotype) could affect *SAA* gene expression. In M1 cells, *SAA1* expression was slightly induced (Ct 40 to 34) whereas no induction was seen in M2 cells. Interestingly, in M1 cells in the presence of LPS-Dex the upregulation of *SAA1* and *SAA2* expression was further enhanced as compared to non-polarized monocytes (Ct from 28 to 26; and from 33 to 30, respectively) (S4 Fig). A moderate increase in the expression of *SAA4* in M1 cells treated with LPS-Dex was also observed (Ct from 40 to 33). In M2 cells, the effect of LPS-Dex on *SAA1* mRNA expression was less pronounced as compared to LPS-Dex treated cells (three donors tested) (S4 Fig). In M1 cells the mRNA expression of *IL1B* was slightly up-regulated, whereas an important increase was observed in the presence of LPS-Dex. The effect of LPS-Dex was decreased in M2 cells as compared to M1 or non-polarized cells (S4 Fig).

### SAA and IL1β secretion by human monocytes

In contrast to mRNA expression, secreted SAA1 levels were always below the ELISA detection threshold (~1.5 ng/mL), in supernatants of cells treated with LPS or LPS-Dex or in supernatants of non-treated cells.

**Fig 2:**
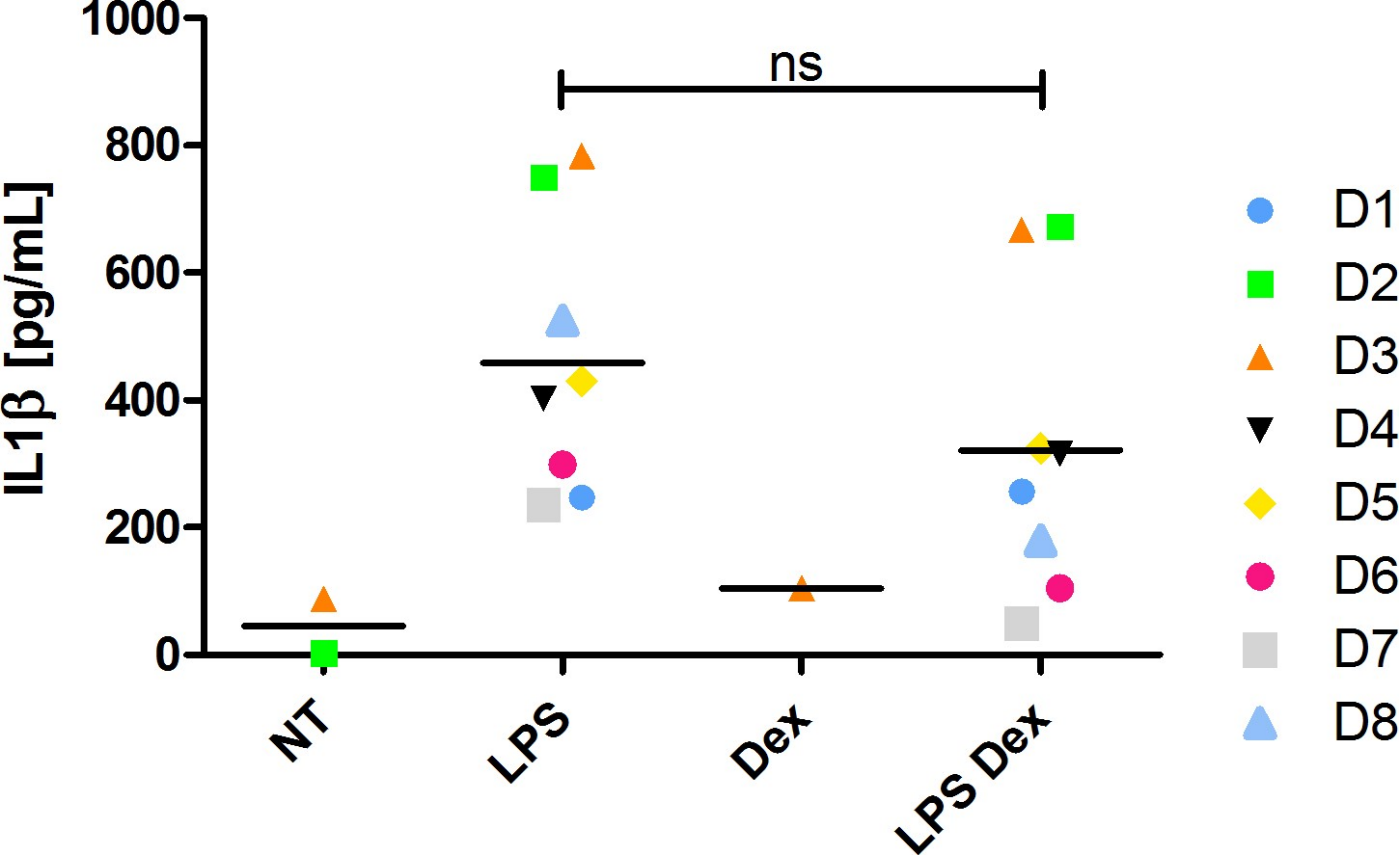
IL1β secretion in human monocyte supernatants. IL1β secretion from monocyte supernatants treated for 24h with 100 ng/ml LPS with or without 1μM Dex from 8 independent donors (D 1–8) as assessed by ELISA. The mean is also represented. Mann Whitney statistical analysis was performed between the two indicated conditions.

However, LPS-induced IL1β secretion followed gene expression. Dexamethasone alone had no effect in IL1β secretion, whereas treatment with LPS-Dex induced a milder secretion of IL1β as compared to LPS alone (Fig 2).

We were unable to detect SAA1 even after supernatant concentration, although under the same conditions IL1β was detected.

### Expression of *SAA1*, *SAA2*, *SAA4* and pro-inflammatory cytokines in human 7-, 14- and 21-day culture macrophages

**Fig 3:**
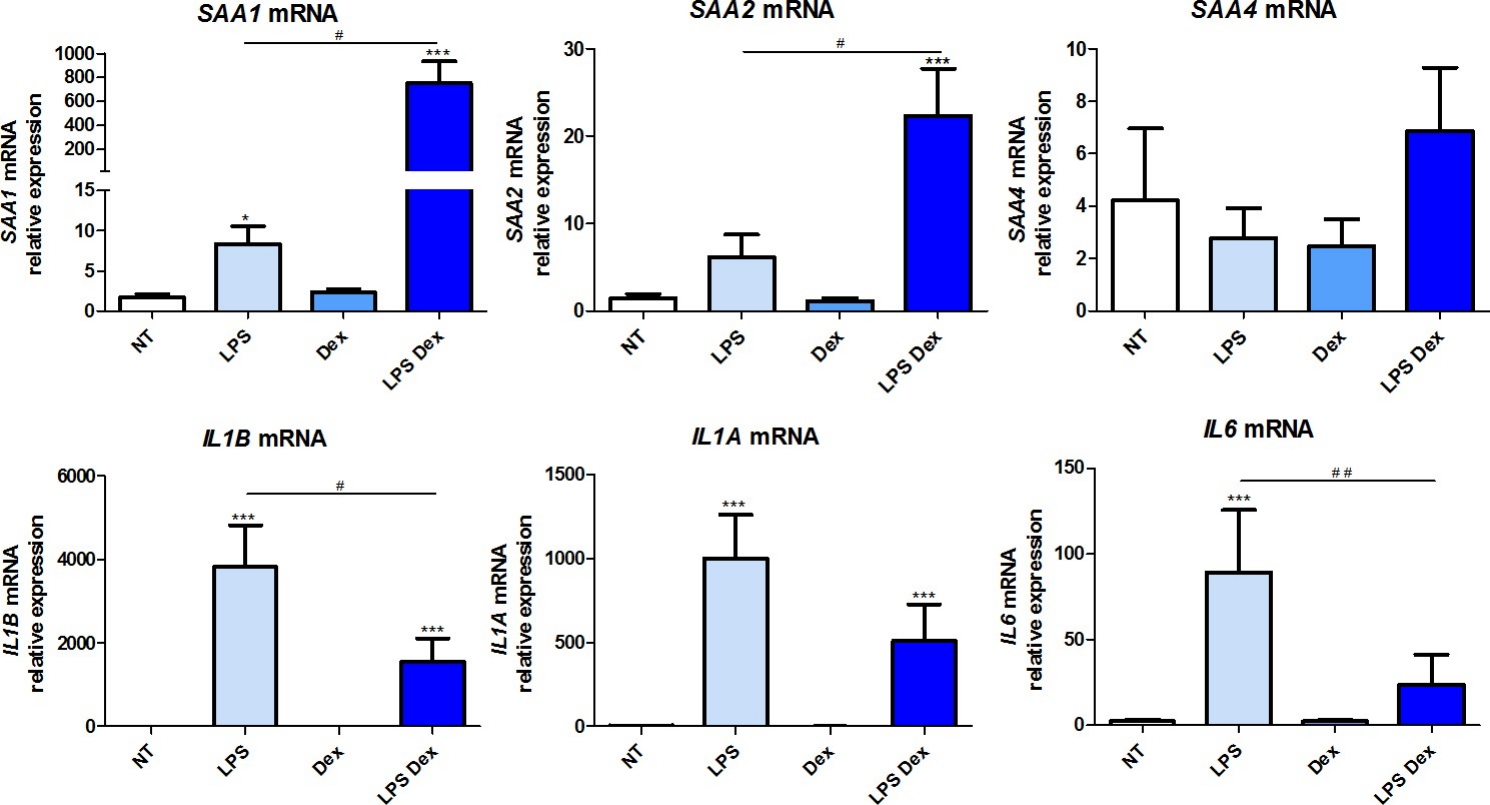
Expression of *SAA1*, *SAA2*, *SAA4* and pro-inflammatory cytokines in human 7-day culture monocyte-derived macrophages. mRNA gene expression was measured by RT-qPCR and presented as relative fold change of non-treated macrophages. Data represent the mean ± standard error of the mean (SEM) of ≥ 5 experiments performed in cells isolated from ≥ 5 independent donors. Asterisks indicate significant differences as compared to non-treated cells (Mann Whitney test: * p < 0.05; ** p < 0.01, *** p<0.001). (#) points out significant differences between the indicated groups (Mann Whitney test: # p < 0.05, ## p< 0.01).

**Fig 4:**
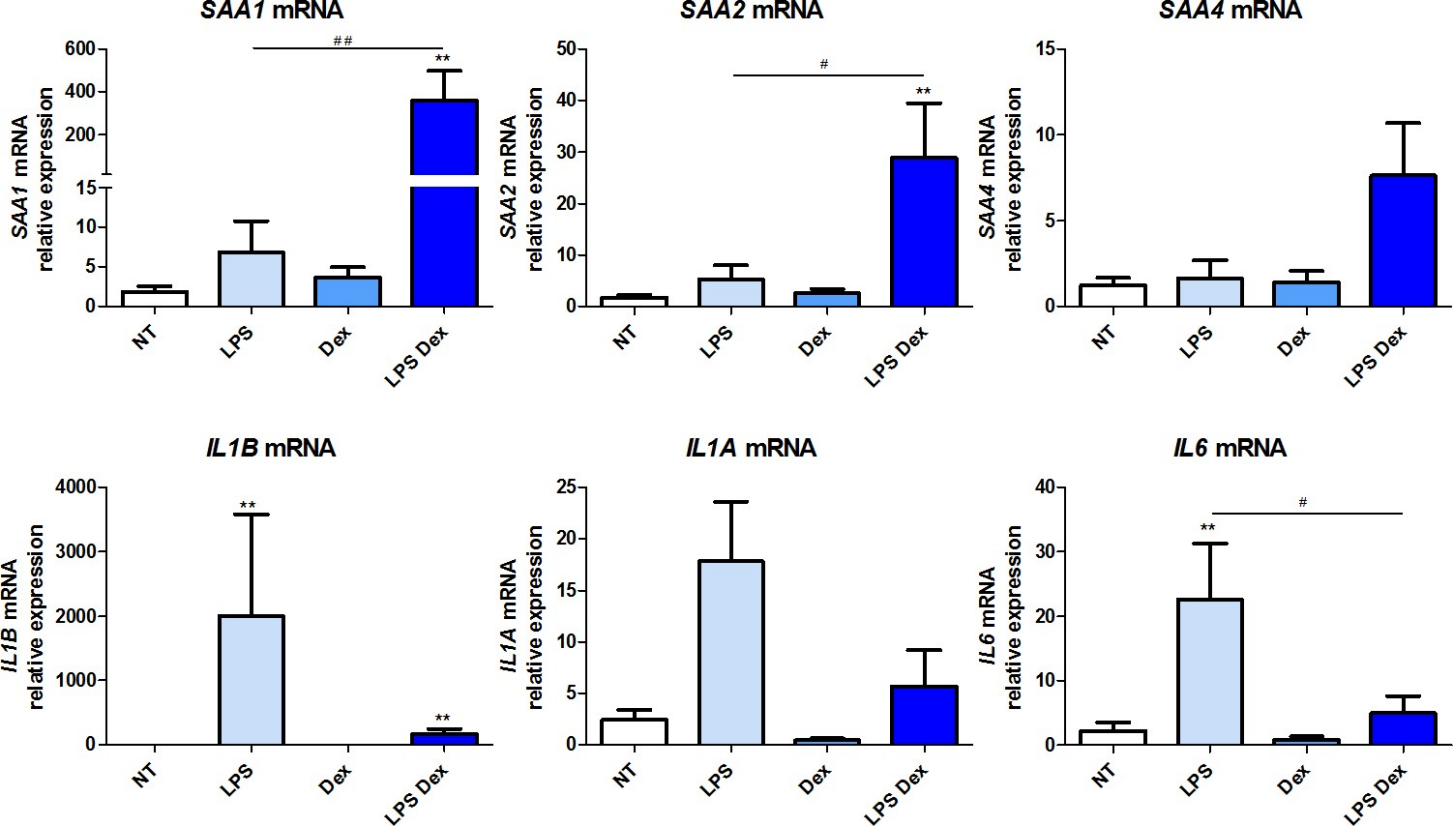
Expression of *SAA1*, *SAA2*, *SAA4* and pro-inflammatory cytokines in human 14-day culture monocyte-derived macrophages. mRNA gene expression was measured by RT-qPCR and presented as relative fold change of non-treated macrophages. Data represent the mean ± standard error of the mean (SEM) of ≥ 4 experiments performed in cells isolated from ≥ 4 independent donors. Asterisks indicate significant differences as compared to non-treated cells (Mann Whitney test: * p < 0.05; ** p < 0.01, *** p<0.001). (#) points out significant differences between the indicated groups (Mann Whitney test: # p < 0.05, ## p< 0.01).

**Fig 5:**
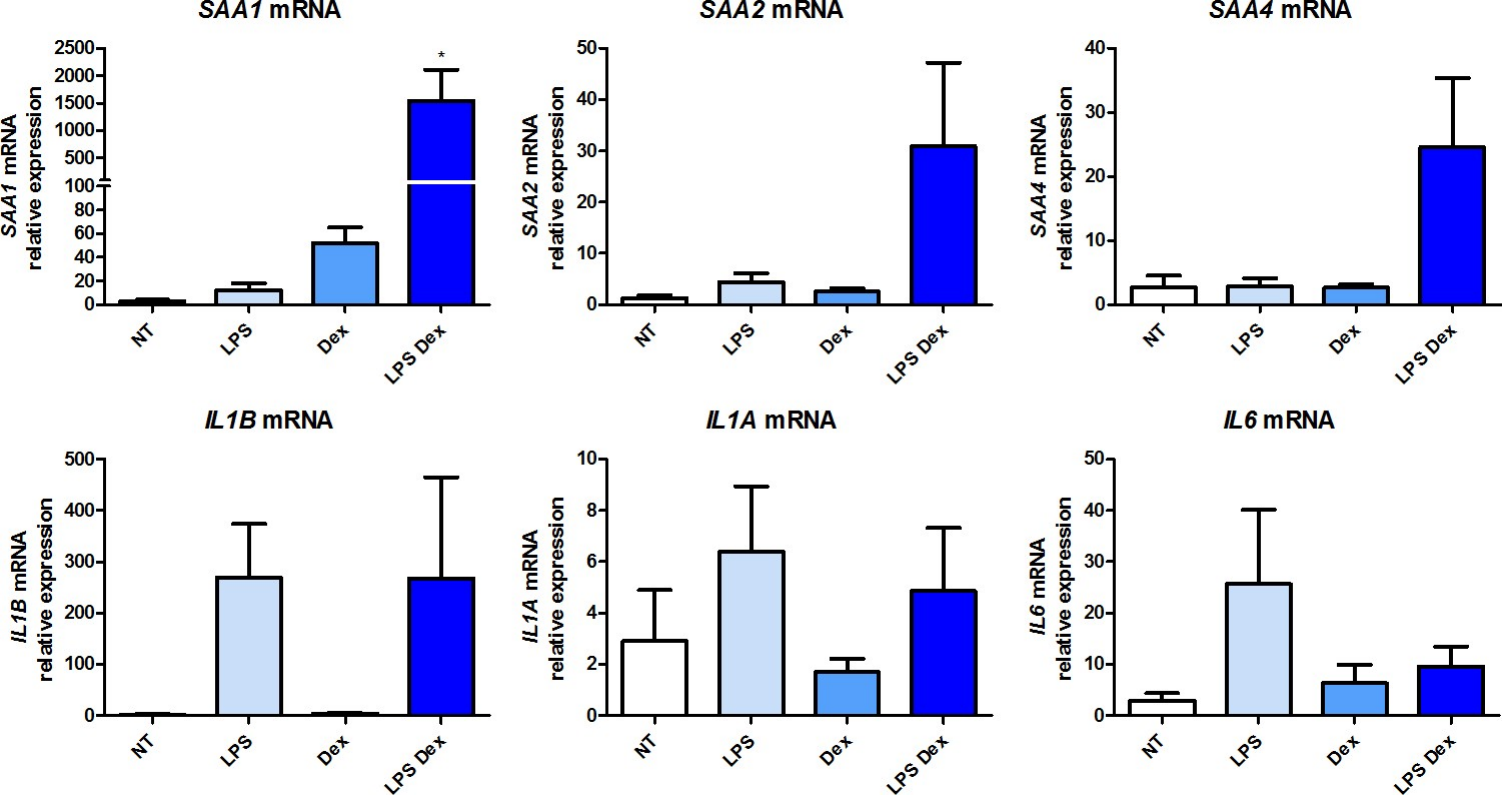
Expression of *SAA1*, *SAA2*, *SAA4* and pro-inflammatory cytokines in human 21-day culture monocyte-derived macrophages. mRNA gene expression was measured by RT-qPCR and presented as relative fold change of non-treated macrophages. Data represent the mean ± standard error of the mean (SEM) of 4 experiments performed in cells isolated from 4 independent donors. Asterisks indicate significant differences as compared to non-treated cells (Mann Whitney test: * p < 0.05; ** p < 0.01, *** p<0.001).

*SAA* gene expression was studied in 7-, 14- and 21-day cultures of human monocyte-derived macrophages. Similarly to monocytes, the basal relative mRNA level of *SAA1*, *SAA2*, *SAA4* was very low (Ct>35) (upper panels of Figs 3–5 respectively). Treatment with LPS alone induced a minor increase in *SAA1* and *SAA2* expression, significant only for *SAA1* in 7-day culture macrophages (p = 0.01). *SAA4* expression was not impacted by LPS treatment. Dex alone did not modify the expression of *SAA1*, *SAA2* or *SAA4*. The combined treatment of LPS-Dex induced a major increase in *SAA1* expression in 7-, 14- and 21-day culture macrophages (p = 0.0002, p = 0.002 and p<0.05, respectively) and to a lesser extent for *SAA2* expression in 7- and 14-day culture macrophages (p = 0.0002 and p = 0.002, respectively) (Figs 3 and 4). A non-significant effect of LPS-Dex on *SAA2* expression was observed in 21-day culture macrophages (Fig 5). A minor, not significant, impact on *SAA4* expression was observed in 14- and 21-day culture macrophages (Figs 4 and 5).

As expected, *IL1B* and *IL6* mRNA expression was significantly induced in the presence of LPS alone in 14- and 21-day culture macrophages. *IL1A* expression was significantly increased in 7-day but to a lesser extent in 14-day culture macrophages. Dexamethasone alone had no effect on cytokine gene expression as compared to their basal levels. The combined treatment LPS-Dex significantly down-regulated the LPS-induced *IL1B* and *IL6* expression in 7-day (p = 0.04 and p = 0.004, respectively) and *IL6* expression in 14-day culture macrophages (p = 0.04) (Figs 3–5 lower panel), while the expression of *IL1A*, at all-time points, *IL1B* in 14- and 21-day and *IL6* in 21-day culture macrophages was reduced, although did not reach statistical significance.

### Effect of methylprednisolone on the expression of *SAA1*, *SAA2*, *SAA4* and pro-inflammatory cytokines in human 7-day culture macrophages

To confirm the impact of glucocorticoids combined to LPS on the expression of *SAA* genes, we comparatively tested methylprednisolone and dexamethasone on 7-day culture macrophages from 4 donors (S5 Fig). Methylprednisolone alone increased the *SAA1* mRNA expression (Ct from 39 to 32, p = 0.02) but had no impact on the expression of *SAA2* and *SAA4*. The combination with LPS and methylprednisolone induced a major *SAA1* expression (Ct from 39 to 25). LPS and methylprednisolone also induced the expression of *SAA2* (Ct from 35 to 30) but the variability among donors was important. *SAA4* expression remained very low (Ct>31).

Similar to LPS-Dex, treatment with LPS-methylprednisolone significantly decreased the LPS-induced mRNA expression of *IL6* and decreased *IL1B* and *IL1A* expression even if the difference between the two conditions did not reach statistical significance due to the variability among the 4 donors.

### Expression of *SAA1*, *SAA2*, *SAA4* in HepG2 cells

HepG2 cells were used as a positive control of SAA expression and secretion. Treatment of HepG2 cells with LPS alone or LPS-Dex had no impact on *SAA* gene expression. Treatment of HepG2 cells with IL1β alone induced *SAA1* expression (Ct from 37 to 30) (S6 Fig). Treatment of HepG2 cells with IL6 induced *SAA1* expression but to a lower extent (Ct from 37 to 33). The combined treatment with IL1β and IL6 greatly increased the *SAA1* expression (Ct from 37 to 24). This effect was more pronounced when HepG2 were treated with Dex concomitantly to IL1β and IL6 (Ct from 37 to 23). Similar effects were observed for *SAA2*, whereas the constitutively expressed *SAA4* was not impacted by the treatment (Ct range from 27–25). SAA1 secretion was measurable (S7A Fig) in the supernatants of HepG2 cells treated with IL1β-IL6 (~17 ng/mL). When cells were treated with IL1β-IL6-Dex (S7A Fig), the secretion was higher (~120 ng/mL) as compared to IL1β-IL6 alone, a result that correlated with the mRNA expression.

### Expression of *SAA1*, *SAA2*, *SAA4* in human adherent monocytes in the presence of cytokines

In selected experiments, monocytes were treated with IL1β and IL6 to mimic conditions that induce the *SAA* expression in HepG2 cells. The combined treatment of IL1β-IL6 had no impact on basal *SAA* gene expression (Ct ~38, either for *SAA1*, *SAA2*, or *SAA4*). The combined treatment of IL1β-IL6-Dex induced *SAA1* and *SAA2* gene expression (from Ct ~38 to ~28 and 34 respectively). However, no SAA1 secretion was measurable under IL1β-IL6-Dex treatment.

### SAA1 protein expression by human monocytes

**Fig 6:**
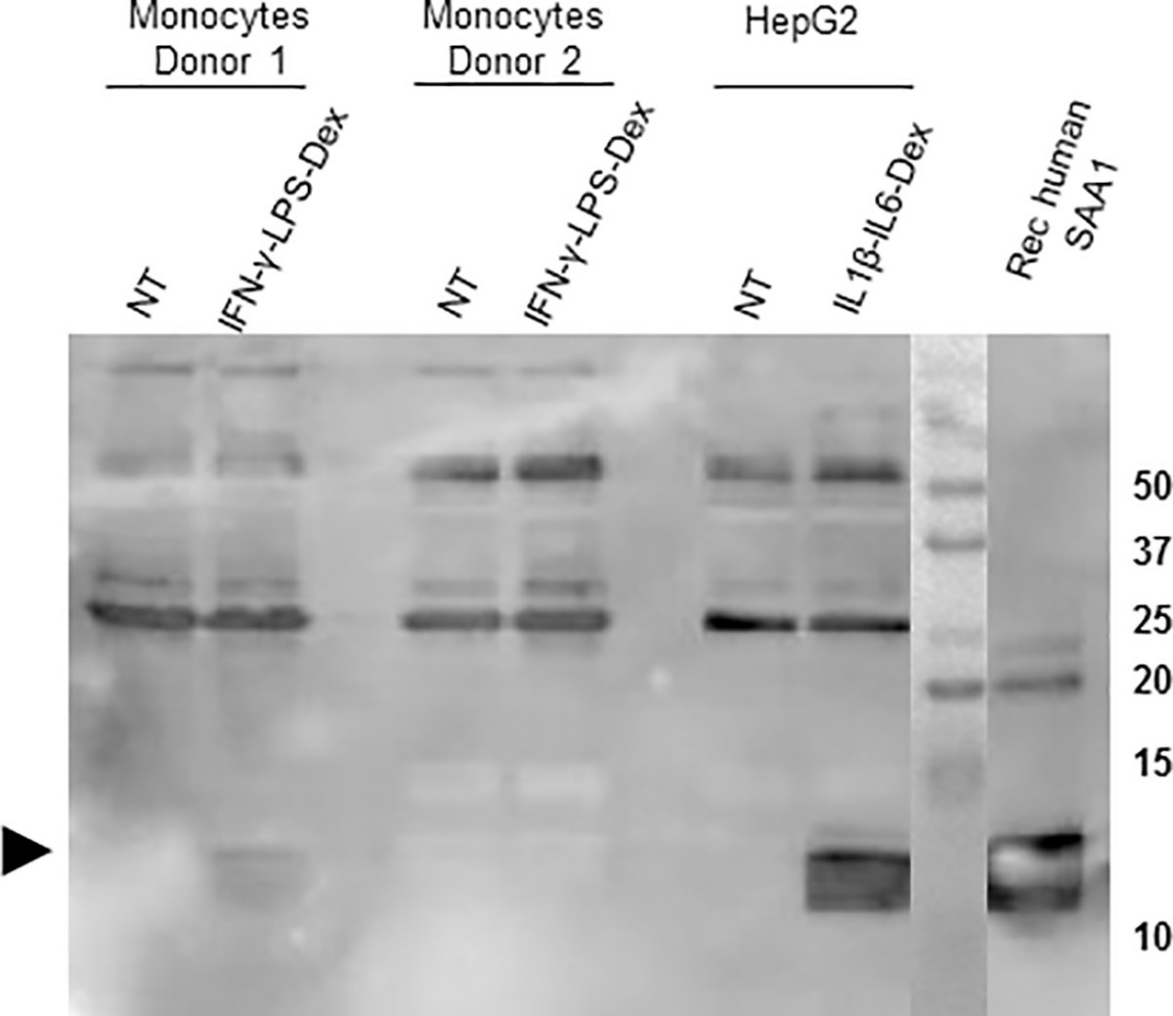
SAA1 protein expression in human monocytes and HepG2. Western blot analysis from immunoprecipitated cell lysates of human monocytes treated or not with IFN-γ-LPS-Dex and HepG2 cells (used as positive control) treated or not with IL1β-IL6-Dex. The figure is representative of six experiments performed in monocytes isolated from six individual buffy coats. A band corresponding to the molecular weight of SAA was detected in monocytes isolated from two out of six donors. For each immunoprecipitation experiment, monocytes were isolated by adherence of 90 x 10^6^ PBMCs as described in methods. Around 5–7 x 10^6^ HepG2 cells were used for each immunoprecipitation. Arrow represents the molecular weight corresponding to SAA1 (12-14KDa). Full size images of the western blot as well as brightness and clearness adjustments are shown in S9 Fig.

Immunoprecipitation experiments were performed in cell lysates of human monocytes treated or not with LPS-Dex (S7B and S8 Figs) or INF-γ-LPS-Dex (Fig 6 and S9 Fig). HepG2 cells treated with IL1β-IL6-Dex were used as a positive control. As shown in Fig 6 and S7B Fig, following immunoprecipitation, the SAA1 protein was detected in HepG2 cells but not in lysates of monocytes treated with LPS-Dex (S7B Fig). However, SAA1 protein was evidenced by immunoprecipitation in monocytes of two out of six healthy donors (Fig 6) after combining INF-γ (M1 phenotype) with LPS-Dex.

## Discussion

Human SAA proteins are important acute phase reactants and precursors of AA amyloidosis and partners of the “cytokine-serum amyloid A-chemokine network” [11]. Despite evidence for extrahepatic SAA production [33], the potential role of human monocytes and macrophages -major players in inflammation- in local SAA expression is still unknown. A difficulty for this evaluation is that human resident macrophages, with the exception of alveolar macrophages, are not easily accessible [50]. In this study, we used human monocytes from peripheral blood, selected by adherence or by CD14-positive selection, and macrophages that were differentiated from monocytes after 7, 14 or 21 days in culture; during this period, cells acquire the morphological and functional characteristics of tissue macrophages, which after 14 days in culture, present an epithelioid cell pattern similar to that of macrophages observed in granulomas [51,52].

In the present study, we clearly show an induction of *SAA1* and *SAA2* gene transcription in monocytes and monocyte-derived macrophages in response to a combined stimulation with LPS and glucocorticoids. As previously reported by others [5,8], no expression of *SAA3* was detected whereas *SAA4* was constitutively expressed at very low levels whatever the conditions used. Our results are in line with those previously reported on human macrophage cell lines showing a *SAA* gene transcription in response to LPS-Dex stimulation [43,45]. However in those studies, detection of *SAA* mRNA was performed using primers that cannot distinguish between *SAA1* and *SAA2* due to the high homology between the two genes [45,53]. For this reason, in the present study, we designed primers that recognize specifically *SAA1* or *SAA2* mRNA. We were therefore able to show that *SAA1* is the major *SAA* acute phase gene induced in human monocytes and derived macrophages in the presence of LPS combined to glucocorticoids. Similar results were obtained either for monocytes selected after 1h adherence or using CD14-positive selection, suggesting that adherence *per se* does not have a significant effect on *SAA* gene expression. In addition, when a pro-inflammatory M1 phenotype was induced by IFN-γ, a potentiation of LPS-Dex effect in inducing *SAA1* gene expression was observed, suggesting that the inflammatory environment modulates *SAA* gene expression.

The acute *SAA* gene induction in the presence of LPS-dexamethasone/methylprednisolone illustrates the pleiotropic roles of glucocorticoids in an inflammatory context [54]. The anti-inflammatory actions of glucocorticoids are regulated mainly through glucocorticoid receptor signaling [55]. However, pro-inflammatory effects of glucocorticoids have also been demonstrated, particularly during the initial phase of activation of the immune system in response to stress signals. For example, it has been shown that in the presence of pro-inflammatory cytokines such as TNFα, glucocorticoids enhance the expression of both *TLR2* mRNA and protein in A549 epithelial cell line [56,57]. In addition, whole genome microarray analysis in the A549 epithelial cell line highlighted 311 genes coregulated by glucocorticoids and TNFα [58]. Among them, *SAA1* and *SAA2* were found to be up-regulated by the synergistic treatment [58]. In such cases, the action of glucocorticoids is considered to prepare the innate immune system for acute response [54].

In the present study, LPS alone was not sufficient to induce *SAA1* and *SAA2* gene expression although it up-regulated the expression of *IL1B*, *IL1A* and *IL6*. LPS is a potent activator of pro-inflammatory gene expression in immune cells, especially in monocytes [59], and although its exact mode of action is not clearly established, transcription factors like nuclear factor-κB (NFκB), nuclear factor-IL-6 (NFIL6) and activator protein-1 (AP-1) have been shown to be involved [60–62]. Despite the presence of NFκB, NFIL6 and AP-1 binding sites on both the *SAA1* and *SAA2* promoters [31,63,64], a combined treatment of LPS with glucocorticoids (dexamethasone or methylprednisolone) was necessary for *SAA* gene induction, as also shown by Yamada et al [45] in the THP1 cell line. Similar results were obtained when, instead of LPS, a combination of glucocorticoids with pro-inflammatory cytokines were used. This supports the hypothesis that LPS-induced pro-inflammatory cytokines may be the necessary co-activators for glucocorticoid actions in *SAA* gene expression. Understanding the role of glucocorticoids in the induction of acute *SAA* gene expression is particularly challenging. It is known that glucocorticoids, after binding to specific cytosolic receptors (GR), act mainly via direct binding to glucocorticoid responsive elements (GRE) present in target gene promoters, or through GR interactions with NFκB and AP-1 [65,66]. A putative GRE has been identified in the *SAA1* promoter whereas the corresponding region in the *SAA2* gene is disrupted by a nine-base insertion [31]. In our studies, glucocorticoids alone were not sufficient to lead to a major increase in *SAA* gene expression in human monocytes or macrophages. Therefore, in agreement with Rao et al., [67], a crosstalk between GR and NFκB pathways may be hypothesized. However, in line with the classical anti-inflammatory role of glucocorticoids [68,69], dexamethasone reduces LPS-induced cytokine expression.

While a combined treatment of LPS-Dex induced the mRNA expression of *SAA1* and to a lesser extent of *SAA2*, we had great difficulty to detect the protein in monocyte- or macrophage-lysates even after immunoprecipitation experiments. Finally, after combining IFN-γ (M1 phenotype) with LPS-Dex, we succeeded in identifying SAA in monocyte lysates. Yet, we were unable to detect SAA secretion even in concentrated monocyte- or macrophage supernatants. However, IL1β secretion measured in parallel was present before and after concentration. The possibility that the levels of SAA were below the detection limit of the ELISA kit (below 1.5 ng/ml) must be considered. Yamada et al. were the only authors reporting SAA secretion by human monocytes-macrophages; it is noteworthy to remind that in order to detect SAA concentrations between 0 to 1.5 ng/mL in monocyte supernatants, they have used an in-house ELISA [45]. One hypothesis is that, even after monocyte-macrophage stimulation, the protein is unstable and quickly degraded. A second hypothesis is that the SAA secretion by monocytes or macrophages is very low and undetectable with the methods used. A third hypothesis is that stimulatory factors present in the human serum may be required to reach a measurable level of SAA; our experiments have been performed in serum-free medium to avoid the effect of SAA internalized from human serum [70]. Yamada et al. described a low SAA secretion related to incubation with elution fractions from fetal calf serum containing IgG and or albumin [45]. However, adding fetal calf serum in the culture medium did not modify our results. The dependence on serum may be specific to some cell types, as in similar experiments with HepG2 cells, we were able to detect SAA secretion in serum-free conditions. However, one should bear in mind that a discrepancy between a low transcriptional activity and high *SAA* mRNA accumulation was previously reported in Hep3B hepatic cell lines [30,71]. Indeed, besides transcriptional and post-transcriptional control of *SAA* gene transcription, there is evidence for a translational regulation in *SAA* transcripts while it is not yet well understood [10,30].

The present study demonstrates that stimulated human monocytes and macrophages can express the *SAA1* and *SAA2* genes, mainly *SAA1*, a result which until now was only shown in macrophage-like cell lines. Therefore, it may be hypothesized that SAA can be locally produced by activated monocytes-macrophages in an inflammatory milieu, even at a very low level. In that context, SAA may activate through autocrine/paracrine stimulation various cells especially monocytes-macrophages via receptors like TLR2/4 or FPR2 [8,11] inducing a SAA-chemokine-cytokine cascade [11]. A low concentration of SAA may favour the production of chemokines as CCL2, CCL3, CXCL8 recruiting more monocytes-macrophages and playing pleiomorphic roles in the immune reaction [8,11]. SAA-stimulated monocytes from patients with Behcet’s disease were capable of promoting Th17 from peripheral CD4+ T cells [28].

Macrophages in inflammatory lesions usually form packed aggregates as in the characteristic granulomas of sarcoidosis [72,73]. SAA has been detected by immunohistochemistry in sarcoidosis granulomas and is suggested to play a role in their maintenance [29,40]. It may be hypothesized that secreted SAA from macrophage-derived cells may reach a significant concentration in the confined intercellular milieu of granulomas and play an autocrine/paracrine role towards the neighbouring cells. This type of stimulation may also lead to a fibrous remodelling of tissues [29,41,74].

In addition, recombinant SAA was shown to have mixed effect on macrophages, inducing both pro- and anti-inflammatory cytokine expression and M2 differentiation markers [75]. It has been suggested that the persistence of this M1/M2 population may contribute to the deleterious remodelling of tissues and persistence of Th17-mediated inflammatory mechanisms [28,75,76].

In conclusion, while previous studies on this topic almost exclusively dealt with cell lines, this study clearly demonstrates that human monocytes and monocyte-derived macrophage express *SAA1* and *SAA2* genes, mainly *SAA1*, when in an inflammatory milieu, especially after M1 differentiation. This encourages us to consider SAA not only as an acute-phase inflammatory mediator secreted by the liver with a general action on innate immune system but also as a cytokine-like mediator secreted by extra-hepatic cells as macrophages and acting locally. The requirement of glucocorticoids (dexamethasone or methylprednisolone) for *in vitro* monocyte-macrophage activation raises questions about the actions of these widely used therapeutic agents in autoinflammatory/autoimmune chronic diseases.

## Supporting information

S1 Appendix LPS and dexamethasone time and concentration dependent experiments in human monocytes and 7-day culture macrophages. (TIF)

S1 Fig Dose-dependent expression of *SAA1*, *SAA2* and *SAA4* in human monocytes and 7-day culture macrophages. mRNA gene expression was measured by RT-qPCR and presented as relative fold change of non-treated monocytes (upper panel) and 7-days macrophages (lower panel). Data represent the mean ± standard error of the mean (SEM) of 4 experiments performed in cells isolated from 4 independent donors. Asterisks indicate significant differences as compared to non-treated cells (Mann Whitney test: * p < 0.05). # indicates significant differences between the two delimited conditions (Mann Whitney test: # p < 0.05).
(TIF)

S2 Fig Kinetics of *SAA1*, *SAA2*, *SAA4* and pro-inflammatory cytokine expression in human monocytes. mRNA gene expression was measured by RT-qPCR and presented as relative fold change of non-treated monocytes. Data represent the mean ± standard error of the mean (SEM) of 4 experiments performed in cells isolated from 4 independent donors. Asterisks indicate significant differences as compared to non-treated cells (Mann Whitney test: * p < 0.05).
(TIF)

S3 Fig *SAA1*, *SAA2*, *SAA4* and pro-inflammatory cytokine expression in human non-adherent monocytes (CD14+ selected). mRNA gene expression was measured by RT-qPCR and presented as relative fold change of non-treated monocytes. Data represent the mean ± standard error of the mean (SEM) of 2 experiments performed in cells isolated from 2 independent donors.
(TIF)

S4 Fig *SAA1*, *SAA2*, *SAA4* and *IL1B* expression in human polarized monocytes. mRNA gene expression was measured by RT-qPCR and presented as relative fold change of non-treated monocytes. Data represent the mean ± standard error of the mean (SEM) of 5 experiments performed in cells isolated from 5 independent donors except for M2 conditions (n = 3). Asterisks indicate significant differences as compared to non-treated cells (Mann Whitney test: * p < 0.05, ** p < 0.01. # points out significant differences between the indicated groups (Mann Whitney test: # p < 0.05, ## p <0.01).
(TIF)

S5 Fig Influence of glucocorticoids on *SAA1*, *SAA2*, *SAA4* and pro-inflammatory cytokine expression in human 7-day culture macrophages. mRNA gene expression was measured by RT-qPCR and presented as relative fold change of non-treated macrophages. Data represent the mean ± standard error of the mean (SEM) of 4 experiments performed in cells isolated from 4 independent donors. Asterisks indicate significant differences as compared to non-treated cells (Mann Whitney test: * p < 0.05). (#) points out significant differences between the indicated groups (Mann Whitney test: # p < 0.05).
(TIF)

S6 Fig *SAA1*, *SAA2*, *SAA4* expression in HepG2 cells. mRNA gene expression was measured by RT-qPCR and presented as relative fold change of non-treated HepG2. Data represent the values of 2 experiments.
(TIF)

S7 Fig SAA1 protein expression in human monocytes and HepG2. **A.** SAA1 secretion from HepG2 cells treated or not for 24h with IL1β-IL6 or IL1β-IL6-Dex as assessed by ELISA. Data represent the mean ± standard error of the mean (SEM) of 3 experiments. Mann Whitney statistical analysis was performed between the non-treated cells and the cells treated with IL1β-IL6-Dex. **B.** Western blot analysis from immunoprecipitated cell lysates of human monocytes treated or not with LPS-Dex and HepG2 cells treated or not with IL1β-IL6-Dex using an anti-SAA antibody. Figure is representative of 2 independent experiments done in monocytes isolated from buffy coats of 2 independent donors. Arrow represents the molecular weight corresponding to SAA1 (12-14KDa). Full size images of the Western blot as well as brightness and clearness adjustments are shown in **S8 Fig**.
(TIF)

S8 Fig Uncropped images of the western blot presented in S7B Fig. (TIF)

S9 Fig Uncropped images of the western blot presented in Fig 6. (TIF)

S1 Table Primer sequences for qPCR. (TIF)

## Abbreviations

Dex: Dexamethasone
HS: Human serum
LPS: Lipopolysaccharide
M1/M2: monocytes differentiated in pro- (M1) or anti- (M2) inflammatory state
SAA: Serum amyloid A
WB: Western blot

## References

[1] Betts JC, Edbrooke MR, Thakker R V, Woo P. The Human Acute-Phase Serum Amyloid A Gene Family: Structure, Evolution and Expression in Hepatoma Cells. Scand J Immunol. 1991 5 4;34(4):471–82. 1656519 10.1111/j.1365-3083.1991.tb01570.x

[2] Steel DM, Sellar GC, Uhlar CM, Simon S, DeBeer FC, Whitehead AS. A constitutively expressed Serum amyloid A protein gene (SAA4) is closely linked to, and shares structural similarities with, an acute-phase Serum amyloid A protein gene (SAA2). Genomics. 1993 5;16(2):447–54. 10.1006/geno.1993.1209 7686132

[3] Sun L, Ye RD. Serum amyloid A1: Structure, function and gene polymorphism. Gene. 2016;583(1):48–57. 10.1016/j.gene.2016.02.044 26945629 PMC5683722

[4] Uhlar CM, Whitehead AS. Serum amyloid A, the major vertebrate acute-phase reactant. Eur J Biochem. 1999 11 18;265(2):501–23. 10504381 10.1046/j.1432-1327.1999.00657.x

[5] Kluve-Beckerman B, Drumm ML, Benson MD. Nonexpression of the human serum amyloid A three (SAA3) gene. DNA Cell Biol. 1991 11;10(9):651–61. 10.1089/dna.1991.10.651 1755958

[6] Larson MA, Wei SH, Weber A, Weber AT, McDonald TL. Induction of human mammary-associated serum amyloid A3 expression by prolactin or lipopolysaccharide. Biochem Biophys Res Commun. 2003;301(4):1030–7. 12589816 10.1016/s0006-291x(03)00045-7

[7] Uhlar CM, Burgess CJ, Sharp PM, Whitehead AS. Evolution of the serum amyloid A (SAA) protein superfamily. Genomics. 1994 1 15;19(2):228–35. 10.1006/geno.1994.1052 8188253

[8] Ye RD, Sun L. Emerging functions of serum amyloid A in inflammation. J Leukoc Biol. 2015 1 12;98(6):923–9. 10.1189/jlb.3VMR0315-080R 26130702 PMC6608020

[9] Marhaug G, Dowton SB. Serum amyloid A: an acute phase apolipoprotein and precursor of AA amyloid. Baillieres Clin Rheumatol. 1994 8;8(3):553–73. 7525085 10.1016/s0950-3579(05)80115-3

[10] De Buck M, Gouwy M, Wang J, Snick J, Opdenakker G, Struyf S, et al Structure and expression of different serum amyloid A (SAA) variants and their concentration-dependent functions during host insults. Curr Med Chem. 2016 6 3;23(17):1725–55. 10.2174/0929867323666160418114600 27087246 PMC5405626

[11] De Buck M, Gouwy M, Wang JM, Van Snick J, Proost P, Struyf S, et al The cytokine-serum amyloid A-chemokine network. Cytokine Growth Factor Rev. 2016 8;30:55–69. 10.1016/j.cytogfr.2015.12.010 26794452 PMC7512008

[12] Banka CL, Yuan T, Beer MC de, Kindy M, Curtiss LK, Beer FC de. Serum amyloid A (SAA): influence on HDL-mediated cellular cholesterol efflux. J Lipid Res. 1995 1 5;36(5):1058–65. 7658153

[13] Benditt EP, Eriksen N. Amyloid protein SAA is associated with high density lipoprotein from human serum. Proc Natl Acad Sci. 1977 9;74(9):4025–8. 10.1073/pnas.74.9.4025 198813 PMC431828

[14] Coetzee GA, Strachan AF, van der Westhuyzen DR, Hoppe HC, Jeenah MS, de Beer FC. Serum amyloid A-containing human high density lipoprotein 3. Density, size, and apolipoprotein composition. J Biol Chem. 1986 7 25;261(21):9644–51. 3525531

[15] Frame NM, Gursky O. Structure of serum amyloid A suggests a mechanism for selective lipoprotein binding and functions: SAA as a hub in macromolecular interaction networks. FEBS Lett. 2016 3 1;590(6):866–79. 10.1002/1873-3468.12116 26918388 PMC4805461

[16] Han CY, Tang C, Guevara ME, Wei H, Wietecha T, Shao B, et al Serum amyloid A impairs the antiinflammatory properties of HDL. J Clin Invest. 2016;126(1):266–81. 10.1172/JCI83475 26642365 PMC4701569

[17] Kluve-Beckerman B, Manaloor J, Liepnieks JJ. Binding, trafficking and accumulation of serum amyloid A in peritoneal macrophages. Scand J Immunol. 2001 4;53(4):393–400. 11285120 10.1046/j.1365-3083.2001.00879.x

[18] Liepnieks JJ, Kluve-Beckerman B, Benson MD. Characterization of amyloid A protein in human secondary amyloidosis: the predominant deposition of serum amyloid A1. Biochim Biophys Acta. 1995 1 25;1270(1):81–6. 7827140 10.1016/0925-4439(94)00076-3

[19] De Buck M, Berghmans N, Pörtner N, Vanbrabant L, Cockx M, Struyf S, et al Serum amyloid A1α induces paracrine IL-8/CXCL8 via TLR2 and directly synergizes with this chemokine via CXCR2 and formyl peptide receptor 2 to recruit neutrophils. J Leukoc Biol. 2015 12;98(6):1049–60. 10.1189/jlb.3A0315-085R 26297794

[20] Eklund KK, Niemi K, Kovanen PT. Immune functions of serum amyloid A. Crit Rev Immunol. 2012;32(4):335–48. 23237509 10.1615/critrevimmunol.v32.i4.40

[21] Gouwy M, De Buck M, Pörtner N, Opdenakker G, Proost P, Struyf S, et al Serum amyloid A chemoattracts immature dendritic cells and indirectly provokes monocyte chemotaxis by induction of cooperating CC and CXC chemokines. Eur J Immunol. 2015;45(1):101–12. 10.1002/eji.201444818 25345597

[22] De Santo C, Arscott R, Booth S, Karydis I, Jones M, Asher R, et al Invariant NKT cells modulate the suppressive activity of Serum Amyloid A-differentiated IL-10-secreting neutrophils. Nat Immunol. 2010 11;11(11):1039–46. 10.1038/ni.1942 20890286 PMC3001335

[23] Sun L, Zhou H, Zhu Z, Yan Q, Wang L, Liang Q, et al Ex vivo and in vitro effect of Serum Amyloid A in the induction of macrophage M2 markers and efferocytosis of apoptotic neutrophils. J Immunol. 2015 5 15;194(10):4891–900. 10.4049/jimmunol.1402164 25870242 PMC4417396

[24] Baranova IN, Vishnyakova TG, Bocharov AV, Kurlander R, Chen Z, Kimelman ML, et al Serum amyloid A binding to CLA-1 (CD36 and LIMPII analogous-1) mediates serum amyloid A protein-induced activation of ERK1/2 and p38 mitogen-activated protein kinases. J Biol Chem. 2005 3 4;280(9):8031–40. 10.1074/jbc.M405009200 15576377

[25] Cheng N, He R, Tian J, Ye PP, Ye RD. Cutting edge: TLR2 is a functional receptor for acute-phase serum amyloid A. J Immunol. 2008 7 1;181(1):22–6. 18566366 10.4049/jimmunol.181.1.22 PMC2464454

[26] Sandri S, Rodriguez D, Gomes E, Monteiro HP, Russo M, Campa A. Is serum amyloid A an endogenous TLR4 agonist? J Leukoc Biol. 2008 5;83(5):1174–80. 10.1189/jlb.0407203 18252871

[27] Lopalco G, Lucherini OM, Vitale A, Talarico R, Lopalco A, Galeazzi M, et al Putative role of Serum Amyloid-A and proinflammatory cytokines as biomarkers for Behcetʼs disease. Medicine (Baltimore). 2015 10;94(42):e1858. 26496336 10.1097/MD.0000000000001858 PMC4620803

[28] Lucherini OM, Lopalco G, Cantarini L, Emmi G, Lopalco A, Venerito V, et al Critical regulation of Th17 cell differentiation by serum amyloid-A signalling in Behcet’s disease. Immunol Lett. 2018 9 1;201:38–44. 10.1016/j.imlet.2018.10.013 30385329

[29] Chen ES, Song Z, Willett MH, Heine S, Yung RC, Liu MC, et al Serum amyloid A regulates granulomatous inflammation in sarcoidosis through Toll-like receptor-2. Am J Respir Crit Care Med. 2010 2 15;181(4):360–73. 10.1164/rccm.200905-0696OC 19910611 PMC2822973

[30] Sack GH. Serum amyloid A–a review. Mol Med [Internet]. 2018 8 30 [cited 2018 Sep 13];24(1). Available from: https://www.ncbi.nlm.nih.gov/pmc/articles/PMC6117975/10.1186/s10020-018-0047-0 PMC6117975 30165816

[31] Thorn CF, Whitehead AS. Differential glucocorticoid enhancement of the cytokine-driven transcriptional activation of the human acute phase Serum amyloid A genes, SAA1 and SAA2. J Immunol. 2002 1 7;169(1):399–406. 12077270 10.4049/jimmunol.169.1.399

[32] Thorn CF, Lu Z-Y, Whitehead AS. Regulation of the human acute phase serum amyloid A genes by Tumour Necrosis Factor-α, Interleukin-6 and glucocorticoids in hepatic and epithelial cell lines. Scand J Immunol. 2004 2 1;59(2):152–8. 14871291 10.1111/j.0300-9475.2004.01369.x

[33] Urieli-Shoval S, Cohen P, Eisenberg S, Matzner Y. Widespread expression of serum amyloid A in histologically normal human tissues: predominant localization to the epithelium. J Histochem Cytochem. 1998 12 1;46(12):1377–84. 10.1177/002215549804601206 9815279

[34] Kumon Y, Suehiro T, Hashimoto K, Sipe JD. Dexamethasone, but not IL-1 alone, upregulates acute-phase serum amyloid A gene expression and production by cultured human aortic smooth muscle cells. Scand J Immunol. 2001 1 1;53(1):7–12. 11169201 10.1046/j.1365-3083.2001.00829.x

[35] Meek RL, Urieli-Shoval S, Benditt EP. Expression of apolipoprotein serum amyloid A mRNA in human atherosclerotic lesions and cultured vascular cells: implications for serum amyloid A function. Proc Natl Acad Sci. 1994 4 12;91(8):3186–90. 10.1073/pnas.91.8.3186 8159722 PMC43540

[36] Upragarin N, Landman WJM, Gaastra W, Gruys E. Extrahepatic production of acute phase serum amyloid A. Histol Histopathol. 2005 10;20(4):1295–307. 10.14670/HH-20.1295 16136510

[37] O’Hara R, Murphy EP, Whitehead AS, FitzGerald O, Bresnihan B. Acute-phase serum amyloid A production by rheumatoid arthritis synovial tissue. Arthritis Res. 2000;2(2):142–4. 10.1186/ar78 11062604 PMC17807

[38] Vallon R, Freuler F, Desta-Tsedu N, Robeva A, Dawson J, Wenner P, et al Serum Amyloid A (apoSAA) expression is up-regulated in rheumatoid arthritis and induces transcription of Matrix Metalloproteinases. J Immunol. 2001 2 15;166(4):2801–7. 11160347 10.4049/jimmunol.166.4.2801

[39] Chen ES, Moller DR. Etiologies of sarcoidosis. Clin Rev Allergy Immunol. 2015 8 1;49(1):6–18. 10.1007/s12016-015-8481-z 25771769

[40] Huho A, Foulke L, Jennings T, Koutroumpakis E, Dalvi S, Chaudhry H, et al The role of serum amyloid A staining of granulomatous tissues for the diagnosis of sarcoidosis. Respir Med. 2017 5;126:1–8. 10.1016/j.rmed.2017.03.009 28427539

[41] Patterson KC, Chen ES. The pathogenesis of pulmonary sarcoidosis and implications for treatment. Chest. 2018 6;153(6):1432–42. 10.1016/j.chest.2017.11.030 29224832

[42] Bozinovski S, Uddin M, Vlahos R, Thompson M, McQualter JL, Merritt A-S, et al Serum amyloid A opposes lipoxin A₄ to mediate glucocorticoid refractory lung inflammation in chronic obstructive pulmonary disease. Proc Natl Acad Sci. 2012 1 17;109(3):935–40. 10.1073/pnas.1109382109 22215599 PMC3271884

[43] Urieli-Shoval S, Meek RL, Hanson RH, Eriksen N, Benditt EP. Human serum amyloid A genes are expressed in monocyte/macrophage cell lines. Am J Pathol. 1994 9;145(3):650–60. 8080047 PMC1890325

[44] Yamada T, Kakihara T, Kamishima T, Fukuda T, Kawai T. Both acute phase and constitutive serum amyloid A are present in atherosclerotic lesions. Pathol Int. 1996 10;46(10):797–800. 8916152 10.1111/j.1440-1827.1996.tb03552.x

[45] Yamada Wada, Itoh Igari. Serum Amyloid A secretion from monocytic leukaemia cell line THP-1 and cultured human peripheral monocytes. Scand J Immunol. 2000 7 1;52(1):7–12. 10886778 10.1046/j.1365-3083.2000.00734.x

[46] Meek RL, Eriksen N, Benditt EP. Murine serum amyloid A3 is a high density apolipoprotein and is secreted by macrophages. Proc Natl Acad Sci. 1992 9 1;89(17):7949–52. 10.1073/pnas.89.17.7949 1518819 PMC49832

[47] Pégorier S, Stengel D, Durand H, Croset M, Ninio E. Oxidized phospholipid: POVPC binds to platelet-activating-factor receptor on human macrophages. Implications in atherosclerosis. Atherosclerosis. 2006 10;188(2):433–43. 10.1016/j.atherosclerosis.2005.11.015 16386258

[48] Awad F, Assrawi E, Jumeau C, Georgin-Lavialle S, Cobret L, Duquesnoy P, et al Impact of human monocyte and macrophage polarization on NLR expression and NLRP3 inflammasome activation. PLoS ONE. 2017;12(4). 10.1371/journal.pone.0175336 PMC5389804 28403163

[49] Berrebi D, Bruscoli S, Cohen N, Foussat A, Migliorati G, Bouchet-Delbos L, et al Synthesis of glucocorticoid-induced leucine zipper (GILZ) by macrophages: an anti-inflammatory and immunosuppressive mechanism shared by glucocorticoids and IL-10. Blood. 2003 1 15;101(2):729–38. 10.1182/blood-2002-02-0538 12393603

[50] Bernaudin JF, Yamauchi K, Wewers MD, Tocci MJ, Ferrans VJ, Crystal RG. Demonstration by in situ hybridization of dissimilar IL-1 beta gene expression in human alveolar macrophages and blood monocytes in response to lipopolysaccharide. J Immunol. 1988 6 1;140(11):3822–9. 3259599

[51] Bainton DR, Golde DW. Differentiation of macrophages from normal human bone marrow in liquid culture. Electron microscopy and cytochemistry. J Clin Invest. 1978 6;61(6):1555–69. 10.1172/JCI109076 659615 PMC372682

[52] Cohn ZA, Benson B. The differentiation of mononuclear phagocytes: morphology, cytochemistry, and biochemistry. J Exp Med. 1965 1 1;121(1):153–70. 14253481 10.1084/jem.121.1.153 PMC2137972

[53] Couderc E, Morel F, Levillain P, Buffière-Morgado A, Camus M, Paquier C, et al Interleukin-17A-induced production of acute serum amyloid A by keratinocytes contributes to psoriasis pathogenesis. PLoS ONE. 2017;12(7):e0181486 10.1371/journal.pone.0181486 28708859 PMC5510841

[54] Busillo JM, Cidlowski JA. The five Rs of glucocorticoid action during inflammation: ready, reinforce, repress, resolve, and restore. Trends Endocrinol Metab. 2013 3;24(3):109–19. 10.1016/j.tem.2012.11.005 23312823 PMC3667973

[55] Beck IME, van den Berghe W, Vermeulen L, Yamamoto KR, Haegeman G, Bosscher KD. Crosstalk in inflammation: the interplay of glucocorticoid receptor-based mechanisms and kinases and phosphatases. Endocr Rev. 2009 12;30(7):830–82. 10.1210/er.2009-0013 19890091 PMC2818158

[56] Chinenov Y, Rogatsky I. Glucocorticoids and the innate immune system: crosstalk with the Toll-like receptor signaling network. Mol Cell Endocrinol. 2007 9 15;275(1):30–42. 17576036 10.1016/j.mce.2007.04.014

[57] Hermoso MA, Matsuguchi T, Smoak K, Cidlowski JA. Glucocorticoids and Tumor Necrosis Factor alpha cooperatively regulate Toll-like receptor 2 gene expression. Mol Cell Biol. 2004 6;24(11):4743–56. 10.1128/MCB.24.11.4743-4756.2004 15143169 PMC416411

[58] Lannan EA, Galliher-Beckley AJ, Scoltock AB, Cidlowski JA. Proinflammatory actions of glucocorticoids: glucocorticoids and TNFα coregulate gene expression in vitro and in vivo. Endocrinology. 2012 8 1;153(8):3701–12. 10.1210/en.2012-1020 22673229 PMC3404340

[59] Guha M, Mackman N. LPS induction of gene expression in human monocytes. Cell Signal. 2001 2 1;13(2):85–94. 11257452 10.1016/s0898-6568(00)00149-2

[60] Mackman, Brand K, Edgington TS. Lipopolysaccharide-mediated transcriptional activation of the human tissue factor gene in THP-1 monocytic cells requires both activator protein 1 and nuclear factor kappa B binding sites. J Exp Med. 1991 12 1;174(6):1517–26. 10.1084/jem.174.6.1517 1744583 PMC2119026

[61] Natsuka S, Akira S, Nishio Y, Hashimoto S, Sugita T, Isshiki H, et al Macrophage differentiation-specific expression of NF-IL6, a transcription factor for interleukin-6. Blood. 1992 1 15;79(2):460–6. 1730090

[62] Shakhov AN, Collart MA, Vassalli P, Nedospasov SA, Jongeneel CV. Kappa B-type enhancers are involved in lipopolysaccharide-mediated transcriptional activation of the tumor necrosis factor alpha gene in primary macrophages. J Exp Med. 1990 1 1;171(1):35–47. 10.1084/jem.171.1.35 2104921 PMC2187654

[63] Ghosh S, May MJ, Kopp EB. NF-κB AND REL PROTEINS: Evolutionarily Conserved Mediators of Immune Responses. Annu Rev Immunol. 1998 4;16(1):225–60. 9597130 10.1146/annurev.immunol.16.1.225

[64] Poli V. The role of C/EBP isoforms in the control of inflammatory and native immunity functions. J Biol Chem. 1998 11 6;273(45):29279–82. 10.1074/jbc.273.45.29279 9792624

[65] Ayroldi E, Riccardi C. Glucocorticoid-induced leucine zipper (GILZ): a new important mediator of glucocorticoid action. FASEB J. 2009 1 11;23(11):3649–58. 10.1096/fj.09-134684 19567371

[66] Barnes PJ. Anti-inflammatory actions of glucocorticoids: molecular mechanisms. Clin Sci. 1998 6;94(6):557–72. 9854452 10.1042/cs0940557

[67] Rao NAS, McCalman MT, Moulos P, Francoijs K-J, Chatziioannou A, Kolisis FN, et al Coactivation of GR and NFKB alters the repertoire of their binding sites and target genes. Genome Res. 2011 9 1;21(9):1404–16. 10.1101/gr.118042.110 21750107 PMC3166826

[68] Cain DW, Cidlowski JA. Immune regulation by glucocorticoids. Nat Rev Immunol. 2017 2 13;17:233–47. 10.1038/nri.2017.1 28192415 PMC9761406

[69] King EM, Chivers JE, Rider CF, Minnich A, Giembycz MA, Newton R. Glucocorticoid repression of inflammatory gene expression shows differential responsiveness by transactivation- and transrepression-dependent mechanisms. PLoS ONE. 2013 1 14;8(1):e53936 10.1371/journal.pone.0053936 23349769 PMC3545719

[70] Ishii W, Liepnieks JJ, Yamada T, Benson MD, Kluve-Beckerman B. Human SAA1-derived amyloid deposition in cell culture: a consistent model utilizing human peripheral blood mononuclear cells and serum-free medium. Amyloid. 2013 6 1;20(2):61–71. 10.3109/13506129.2013.775941 23461622

[71] Jiang SL, Samols D, Rzewnicki D, Macintyre SS, Greber I, Sipe J, et al Kinetic modeling and mathematical analysis indicate that acute phase gene expression in Hep 3B cells is regulated by both transcriptional and posttranscriptional mechanisms. J Clin Invest. 1995 3 1;95(3):1253–61. 10.1172/JCI117775 7883974 PMC441464

[72] Valeyre D, Prasse A, Nunes H, Uzunhan Y, Brillet P-Y, Müller-Quernheim J. Sarcoidosis. The Lancet. 2014 3 29;383(9923):1155–67. 10.1016/S0140-6736(13)60680-7 24090799

[73] Soler P, Basset F, Bernaudin JF, Chretien J. Morphology and distribution of the cells of a sarcoid granuloma: ultrastructural study of serial sections. Ann N Y Acad Sci. 1976;278(1):147–60. 1067004 10.1111/j.1749-6632.1976.tb47026.x

[74] Chen ES, Moller DR. Sarcoidosis—scientific progress and clinical challenges. Nat Rev Rheumatol. 2011 8;7(8):457–67. 10.1038/nrrheum.2011.93 21750528

[75] Anthony D, McQualter JL, Bishara M, Lim EX, Yatmaz S, Seow HJ, et al SAA drives proinflammatory heterotypic macrophage differentiation in the lung via CSF-1R-dependent signaling. FASEB J. 2014 9;28(9):3867–77. 10.1096/fj.14-250332 24846388 PMC5395724

[76] Beringer A, Miossec P. IL-17 and IL-17-producing cells and liver diseases, with focus on autoimmune liver diseases. Autoimmun Rev. 2018 12 1;17(12):1176–85. 10.1016/j.autrev.2018.06.008 30321671

