# Supplementary figures and images for: Expression of SAA1, SAA2 and SAA4 genes in human primary monocytes and monocyte-derived macrophages
Source: PLoS One. 2019 May 17;14(5):e0217005. doi: 10.1371/journal.pone.0217005 (PMC6524798; doi:10.1371/journal.pone.0217005)

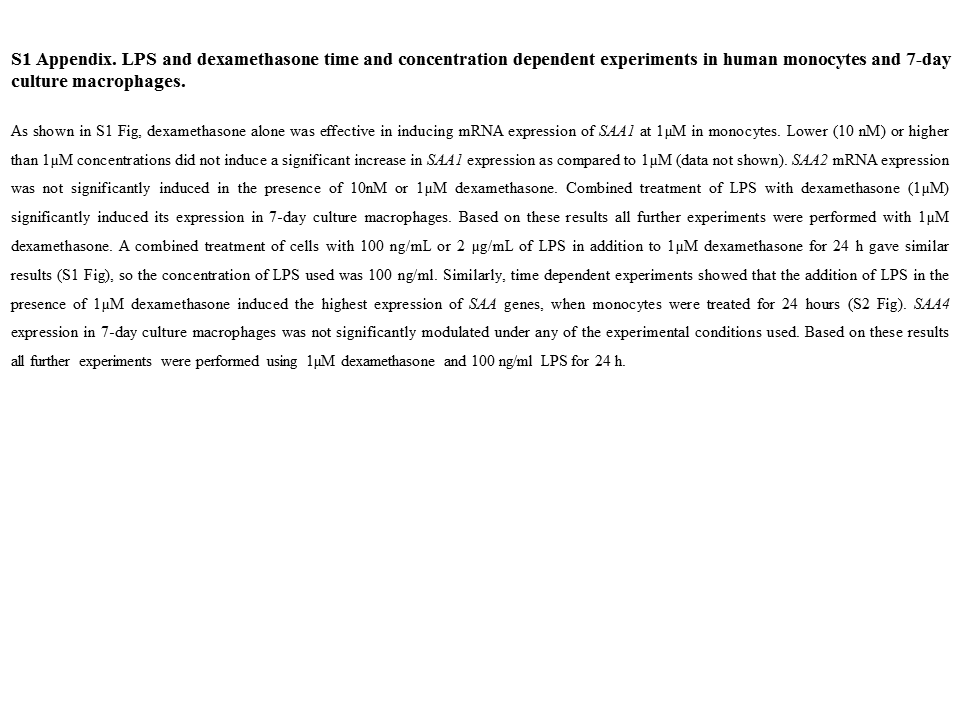

Supplement: S1 Appendix — (TIF) [file pone.0217005.s001.tif]

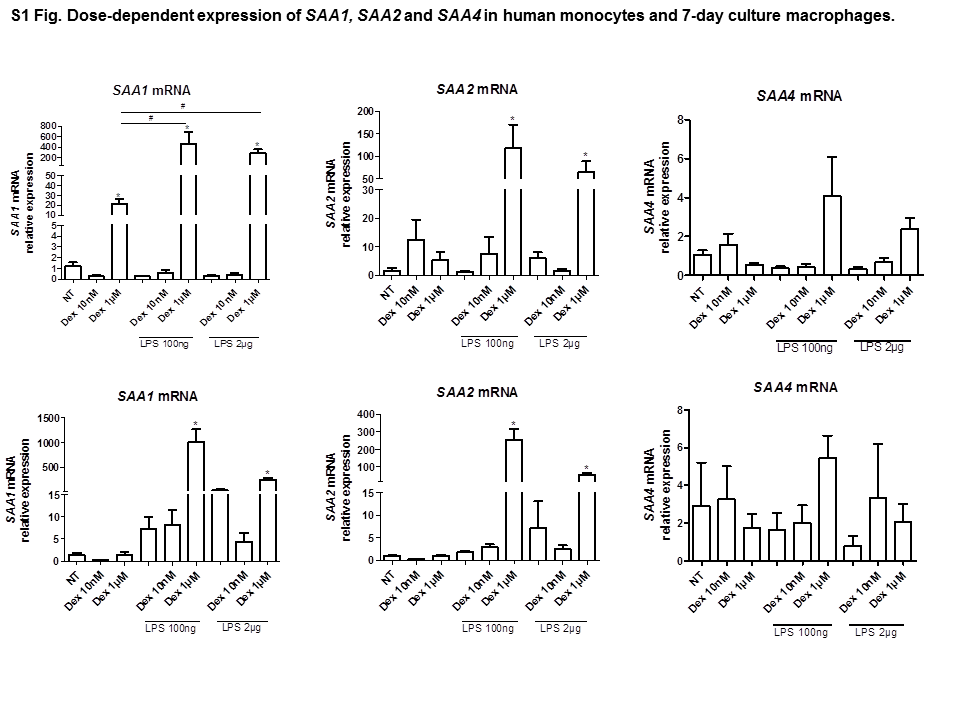

Supplement: S1 Fig — mRNA gene expression was measured by RT-qPCR and presented as relative fold change of non-treated monocytes (upper panel) and 7-days macrophages (lower panel). Data represent the mean ± standard error of the mean (SEM) of 4 experiments performed in cells isolated from 4 independent donors. Asterisks indicate significant differences as compared to non-treated cells (Mann Whitney test: * p < 0.05). # indicates significant differences between the two delimited conditions (Mann Whitney test: # p < 0.05). (TIF) [file pone.0217005.s002.tif]

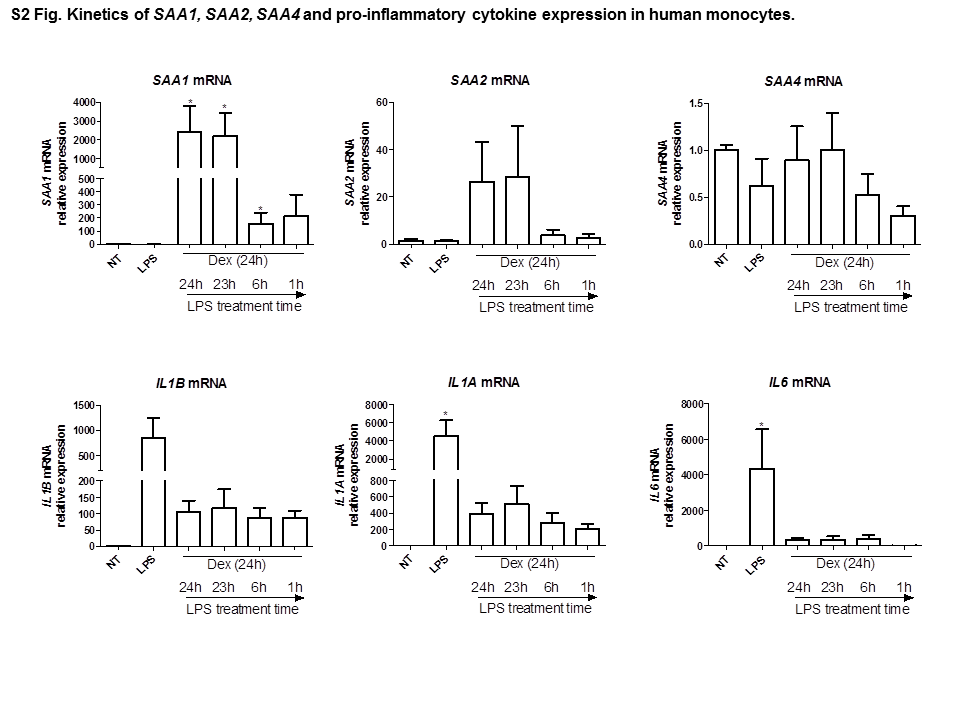

Supplement: S2 Fig — mRNA gene expression was measured by RT-qPCR and presented as relative fold change of non-treated monocytes. Data represent the mean ± standard error of the mean (SEM) of 4 experiments performed in cells isolated from 4 independent donors. Asterisks indicate significant differences as compared to non-treated cells (Mann Whitney test: * p < 0.05). (TIF) [file pone.0217005.s003.TIF]

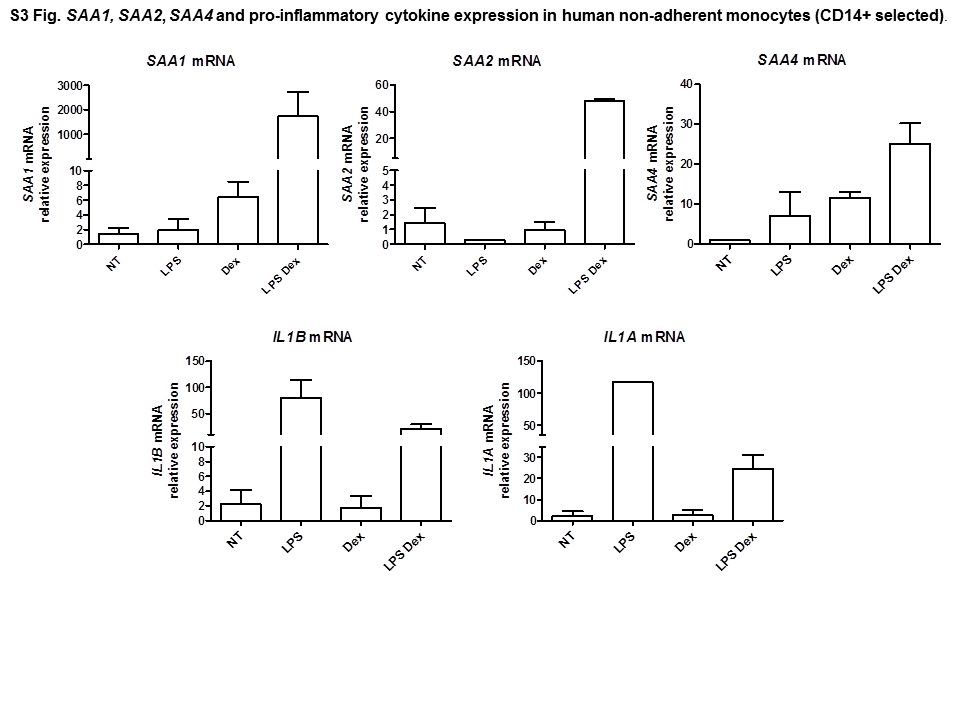

Supplement: S3 Fig — mRNA gene expression was measured by RT-qPCR and presented as relative fold change of non-treated monocytes. Data represent the mean ± standard error of the mean (SEM) of 2 experiments performed in cells isolated from 2 independent donors. (TIF) [file pone.0217005.s004.TIF]

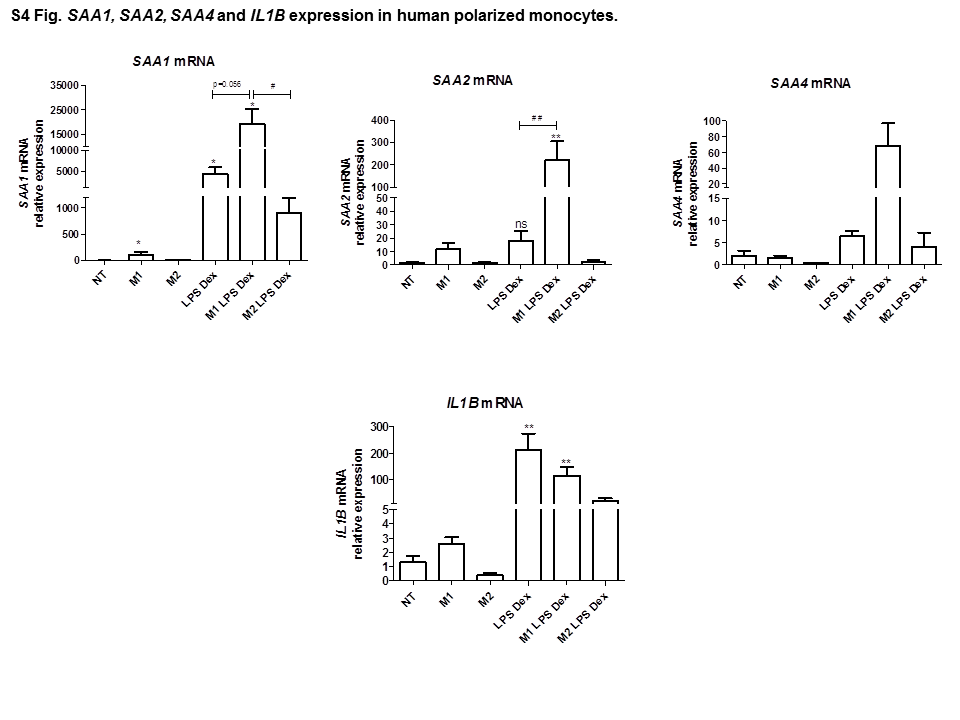

Supplement: S4 Fig — mRNA gene expression was measured by RT-qPCR and presented as relative fold change of non-treated monocytes. Data represent the mean ± standard error of the mean (SEM) of 5 experiments performed in cells isolated from 5 independent donors except for M2 conditions (n = 3). Asterisks indicate significant differences as compared to non-treated cells (Mann Whitney test: * p < 0.05, ** p < 0.01. # points out significant differences between the indicated groups (Mann Whitney test: # p < 0.05, ## p <0.01). (TIF) [file pone.0217005.s005.TIF]

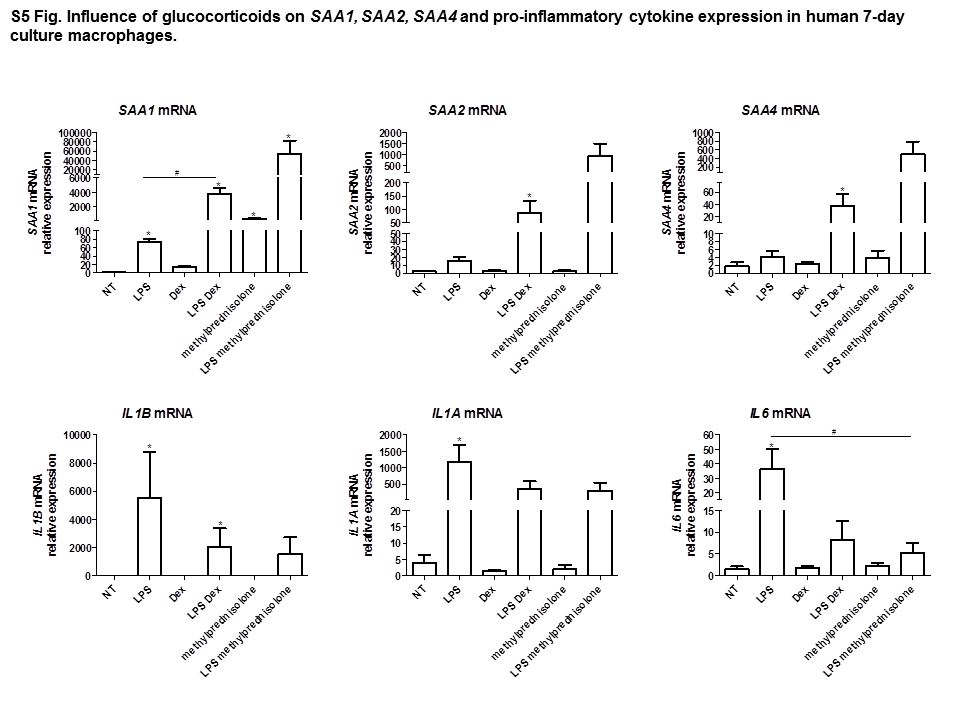

Supplement: S5 Fig — mRNA gene expression was measured by RT-qPCR and presented as relative fold change of non-treated macrophages. Data represent the mean ± standard error of the mean (SEM) of 4 experiments performed in cells isolated from 4 independent donors. Asterisks indicate significant differences as compared to non-treated cells (Mann Whitney test: * p < 0.05). (#) points out significant differences between the indicated groups (Mann Whitney test: # p < 0.05). (TIF) [file pone.0217005.s006.tif]

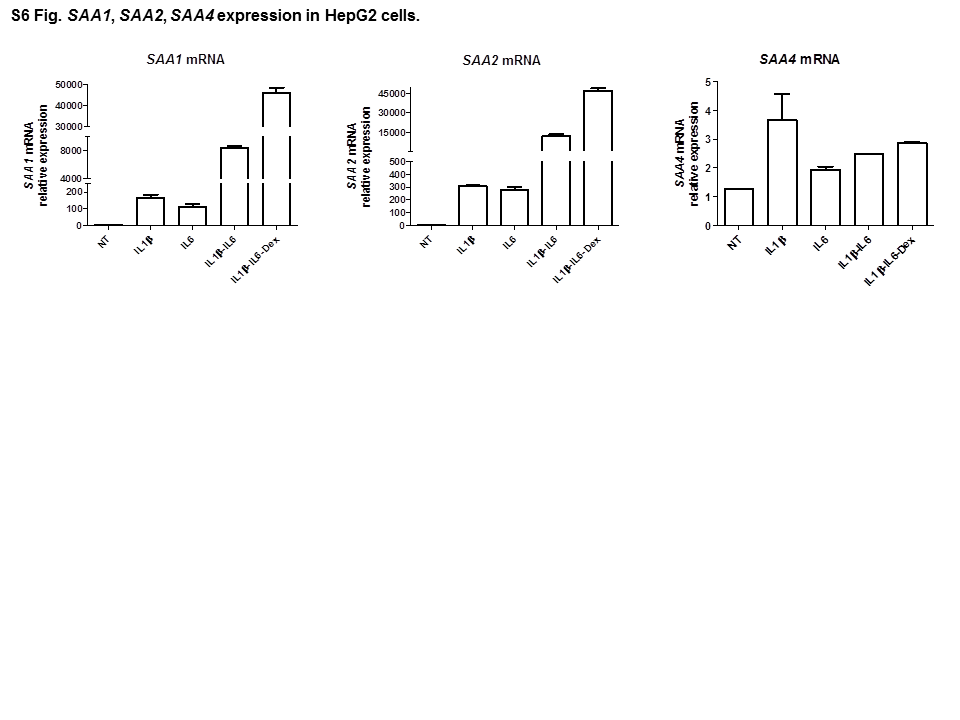

Supplement: S6 Fig — mRNA gene expression was measured by RT-qPCR and presented as relative fold change of non-treated HepG2. Data represent the values of 2 experiments. (TIF) [file pone.0217005.s007.tif]

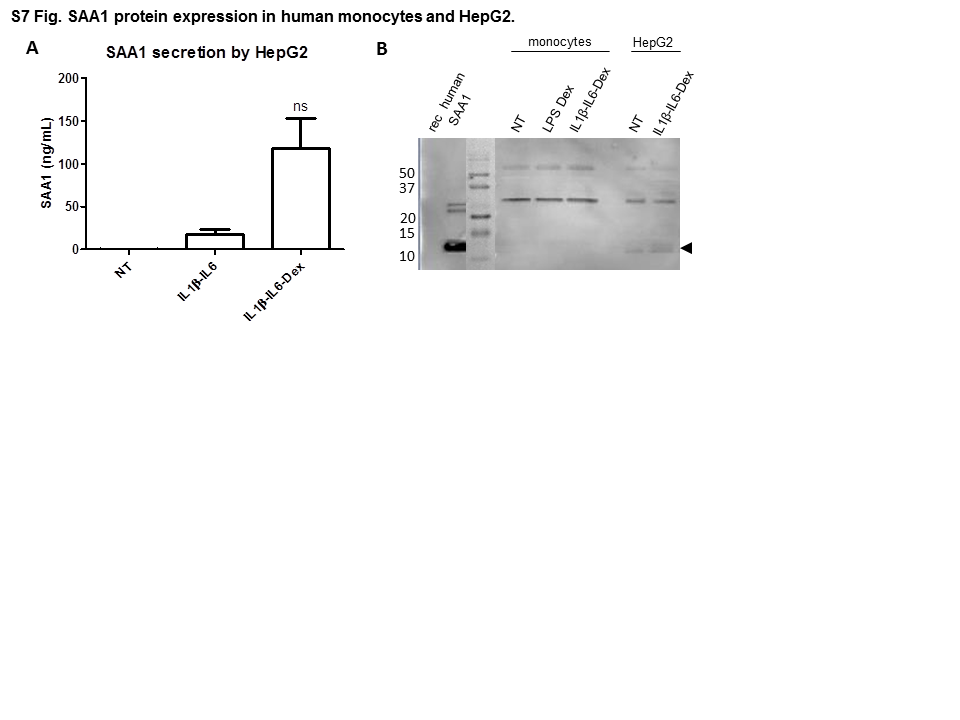

Supplement: S7 Fig — A. SAA1 secretion from HepG2 cells treated or not for 24h with IL1β-IL6 or IL1β-IL6-Dex as assessed by ELISA. Data represent the mean ± standard error of the mean (SEM) of 3 experiments. Mann Whitney statistical analysis was performed between the non-treated cells and the cells treated with IL1β-IL6-Dex. B. Western blot analysis from immunoprecipitated cell lysates of human monocytes treated or not with LPS-Dex and HepG2 cells treated or not with IL1β-IL6-Dex using an anti-SAA antibody. Figure is representative of 2 independent experiments done in monocytes isolated from buffy coats of 2 independent donors. Arrow represents the molecular weight corresponding to SAA1 (12-14KDa). Full size images of the Western blot as well as brightness and clearness adjustments are shown in S8 Fig. (TIF) [file pone.0217005.s008.tif]

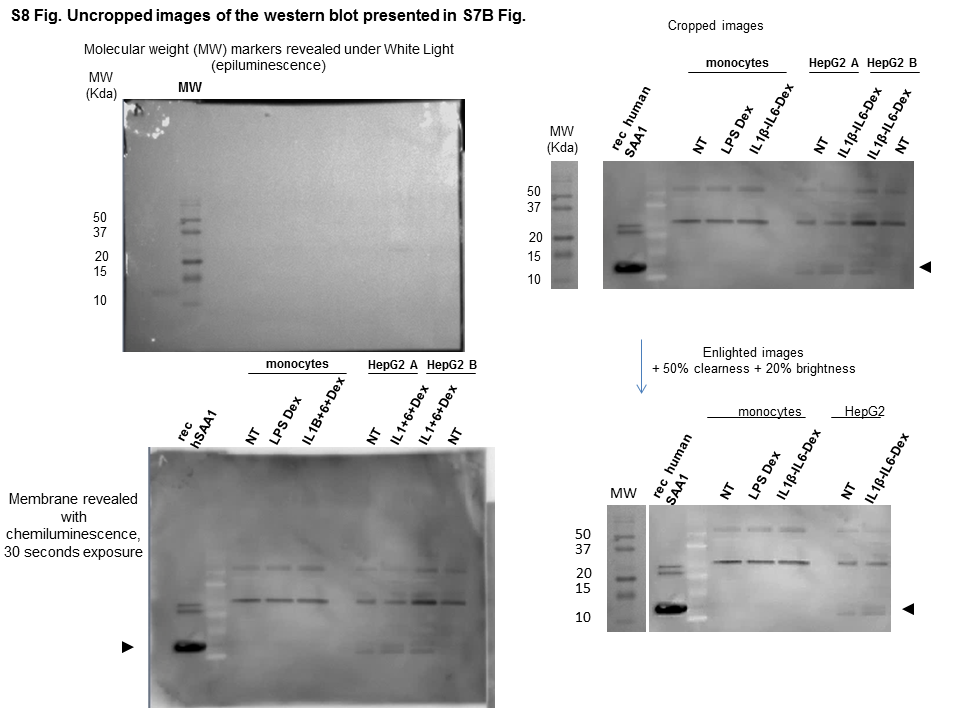

Supplement: S8 Fig — (TIF) [file pone.0217005.s009.tif]

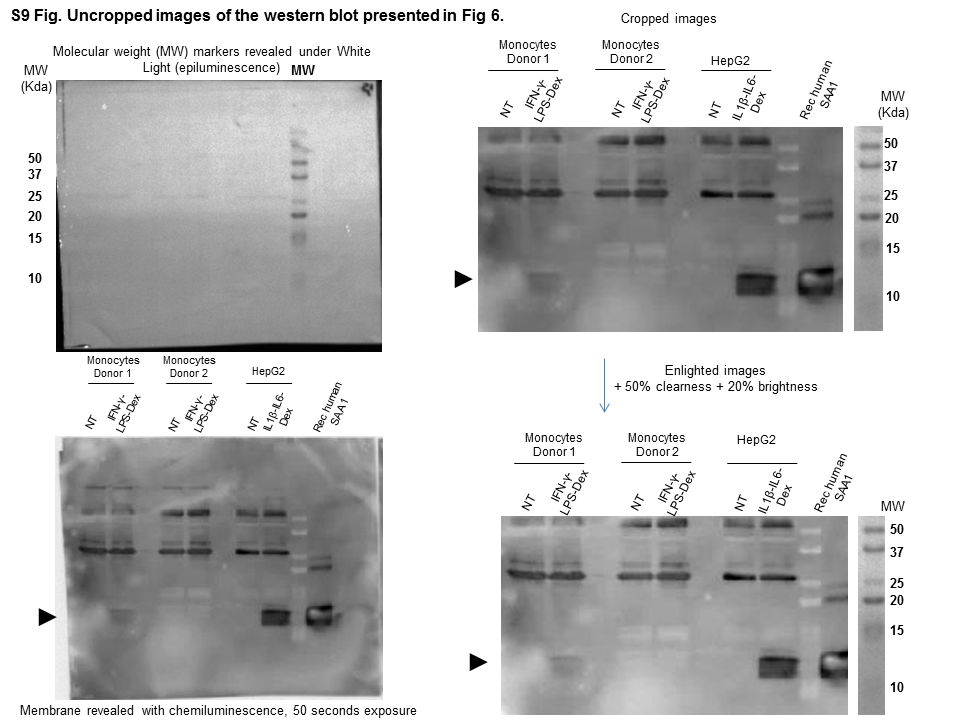

Supplement: S9 Fig — (TIF) [file pone.0217005.s010.tif]

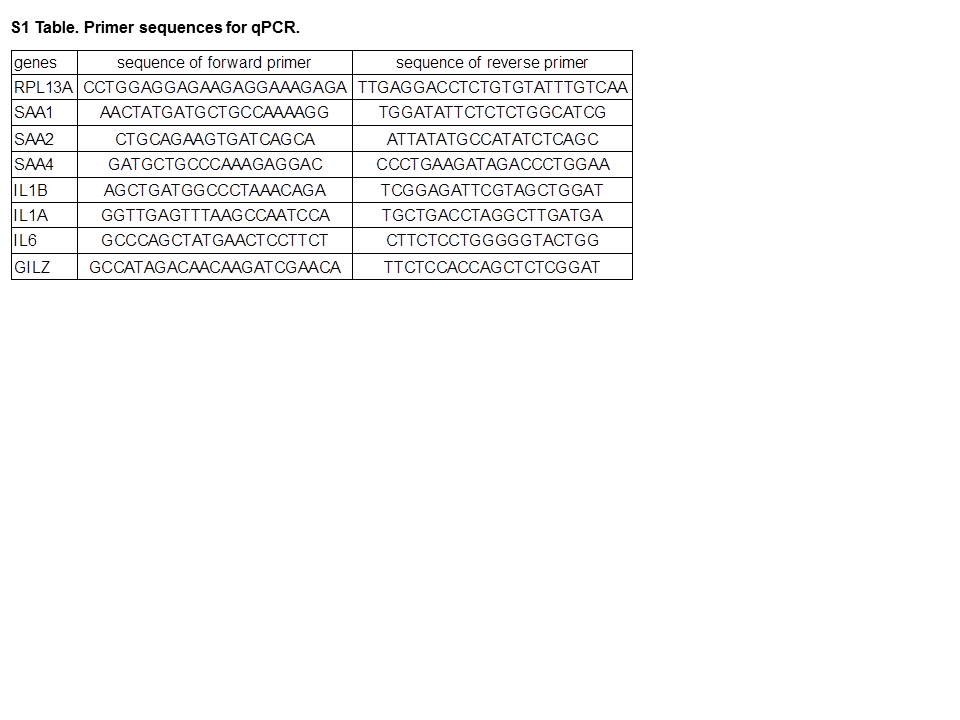

Supplement: S1 Table — (TIF) [file pone.0217005.s011.tif]
